# A sugarcane smut fungus effector simulates the host endogenous elicitor peptide to suppress plant immunity

**DOI:** 10.1111/nph.17835

**Published:** 2021-11-12

**Authors:** Hui Ling, Xueqin Fu, Ning Huang, Zaofa Zhong, Weihua Su, Wenxiong Lin, Haitao Cui, Youxiong Que

**Affiliations:** ^1^ Key Laboratory of Sugarcane Biology and Genetic Breeding Ministry of Agriculture Key Laboratory of Ministry of Education for Genetics, Breeding and Multiple Utilization of Crops Plant Immunity Center College of Life Sciences Fujian Agriculture and Forestry University Fuzhou 350002 China; ^2^ College of Agriculture Yulin Normal University Yulin 537000 China

**Keywords:** effector, PEPR1, plant immunity, smut fungus, sugarcane

## Abstract

- The smut fungus *Sporisorium scitamineum* causes the most prevalent disease on sugarcane. The mechanism of its pathogenesis, especially the functions and host targets of its effector proteins, are unknown.
- In order to identify putative effectors involving in *S. scitamineum* infection, a weighted gene co‐expression network analysis was conducted based on the transcriptome profiles of both smut fungus and sugarcane using a customized microarray. A smut effector gene, termed *SsPele1*, showed strong co‐expression with sugarcane *PLANT ELICITOR PEPTIDE RECEPTOR1* (*ScPEPR1*), which encodes a receptor like kinase for perception of plant elicitor peptide1 (ScPep1). The relationship between SsPele1 and ScPEPR1, and the biological function of SsPele1 were characterized in this study.
- The SsPele1 C‐terminus contains a plant elicitor peptide‐like motif, by which SsPele1 interacts strongly with ScPEPR1. Strikingly, the perception of ScPep1 on ScPEPR1 is competed by SsPele1 association, leading to the suppression of ScPEPR1‐mediated immune responses. Moreover, the *Ustilago maydis* effector UmPele1, an ortholog of SsPele1, promotes fungal virulence using the same strategy.
- This study reveals a novel strategy by which a fungal effector can mimic the plant elicitor peptide to complete its perception and attenuate receptor‐activated immunity.

## Introduction

Sugarcane (*Saccharum* spp. hybrids) is a multifunctional crop especially for sugar production (Marques *et al*., 2017). It is impacted by various diseases, including sugarcane smut, caused by *Sporisorium scitamineum* (Ustilaginomycetes), which occur in the growing areas all over the world (Marques *et al*., 2017). Smut fungi colonize the apical meristematic tissue of the germinating lateral bud or stem apex, resulting in the degradation of plant cell wall, enlargement of the intercellular space, hormonal imbalance and the development of a whip‐like sorus in sugarcane (Marques *et al*., 2017, 2018). The sorus is an elongated internode whose growth is mediated by high mitotic activity of the intercalary meristem at the base (Marques *et al*., 2018). The underlying mechanisms driving the cellular changes in the host tissue remain to be elucidated.

Smut fungi are facultative biotrophs, as they can grow saprotrophically as yeast‐like cells on culture media but require the biotrophic infection of host cells to complete their life cycle (Sundar *et al*., 2012). To establish a biotrophic parasite, these fungi deliver large amounts of effectors to counteract host defenses. *Ustilago maydis* and *Sporisorium reilianum*, which cause smut disease on maize, both produce more than four hundred putative effectors (Schuster *et al*., 2018). Many smut effectors have enzymatic activities, such as mutase, peroxidase or protease (Doehlemann *et al*., 2009, 2011; Djamei *et al*., 2011; Hemetsberger *et al*., 2012; Mueller *et al*., 2013; Ma *et al*., 2018; Schweizer *et al*., 2018). However, many smut‐secreted effectors, accounting for nearly half of the secretome, lack known functional domains (Schuster *et al*., 2018). The *S. scitamineum* genome encodes 622 proteins with signal peptides, among which 537 were predicted as candidate‐secreted effector proteins (Que *et al*., 2014b; Dutheil *et al*., 2016), including the orthologs of well‐studied effectors in *U. maydis*, such as Cmu1, Pep1, Pit2, Stp1 and Tin2 (Tumor inducing2) (Tanaka *et al*., 2014). The transcription of these effector genes was significantly upregulated in the infected tissue (Barnabas *et al*., 2017). However, none of the putative effectors in *S. scitamineum* has been functionally characterized.

Different technologies, including metabolomics (Sánchez‐Elordi *et al*., 2019), proteomics (Barnabas *et al*., 2017) and DNA/RNA related‐omics (Que *et al*., 2014a, 2014b,2014a, 2014b; Su *et al*., 2019), have been used in identifying *S. scitamineum* factors that were involved in sugarcane–*S. scitamineum* interactions. These studies usually focused on either sugarcane or smut fungus, but not on both. Furthermore, most *S. scitamineum* transcripts or proteins are missing from the published sugarcane–*S. scitamineum* mixed transcriptome or proteome data probably due to the low biomass of *S. scitamineum* in the mixed samples. Weighted gene co‐expression network analysis (WGCNA) is a widely used systemic biology method to construct gene networks, detect gene modules and identify the central players within modules (Langfelder & Horvath, 2008). In a co‐expression network between the biomass and gene expression levels in *U. maydis* using WGCNA, three well‐known effector genes *Pep1*, *Pit2* and *Stp1*, and 25 uncharacterized core effector genes were clustered into the same module and were speculated to be important for establishing biotrophy (Lanver *et al*., 2018). An *Arabidopsis thaliana*–*Botrytis cinerea* gene co‐expression network generated using WGCNA revealed that fungal phytotoxins, such as sesquiterpene botrydial and polyketide botcinic acid, likely targeted host defense/camalexin related components to inhibit host immunity (Zhang *et al*., 2019). To date, WGCNA has not yet been used to construct sugarcane–*S*. *scitamineum* gene co‐expression networks and to identify key players in this pathosystem.

The first layer of the plant immune system consists of PAMP‐triggered immunity (PTI) that is activated by cell‐surface‐resident pattern recognition receptors (PRRs), perceiving pathogen/microbe associated‐molecular patterns (PAMPs/MAMPs) or damage‐associated molecular patterns (DAMPs). PTI activates a series of immune responses, including production of reactive oxygen species (ROS) and nitric oxide, phosphorylation of mitogen‐activated protein kinase (MAPK) cascades, transcriptional reprograming, changing of hormone homeostasis, and callose deposition (Cui *et al*., 2015; Liang & Zhou, 2018; Wang *et al*., 2020). PRRs consist primarily of receptor‐like kinases (RLKs) and receptor‐like proteins (RLPs). There are approximately 610 and approximately. 1100 RLKs accounting for *c*. 2% of the coding genes in the Arabidopsis and rice genomes, respectively (Liang & Zhou, 2018). In sugarcane, 427 RLKs and 157 RLPs are coded in the genome of the ancestral sugarcane genotype *Saccharum spontaneum* (Rody *et al*., 2019). RNA‐seq detected 290 RLK and 194 RLP transcripts in modern sugarcane varieties (Rody *et al*., 2019). Among them, 18 were significantly upregulated in a smut‐resistant variety (SP80–3280), whereas only six were upregulated in the smut‐susceptible variety (IAC66–6) (Rody *et al*., 2019), indicating their involvement in resistance to smut disease. However, none of these RLKs or RLPs have been functionally characterized in the sugarcane–*S. scitamineum* interaction thus far.

In this study, using a customized Agilent microarray combined with WGCNA, we discovered *S. scitamineum* putative effectors that exhibit strong co‐expression with a sugarcane *PLANT ELICITOR PEPTIDE RECEPTOR1* (*ScPEPR1*) gene. Plant PEPRs are PRRs recognizing plant elicitor peptides (Peps) that are DAMPs being produced primarily after wounding (Tang & Zhou, 2016). We found that an apoplastic effector, termed SsPele1, interacts with ScPEPR1. SsPele1 has a Peps‐like motif on the C‐terminus and could bind to the ScPEPR1 extracellular LRR domain to compete ligand binding, leading to the suppression of ScPEPR1‐mediated immune responses. The works reveal a novel virulence mechanism of fungal apoplastic effector to suppress host defenses by competing perception of Peps.

## Materials and Methods

### Plant materials, growth conditions and pathogen infection

Sugarcane genotypes, NCo376 (highly resistant to *Sporisorium scitamineum*), YC71‐374 (highly susceptible to *S. scitamineum*) and ROC22 (the most prevalent variety in China) were used. Robust sugarcane plants with uniform growth from NCo376 and YC71‐374 were collected from the field and cut into single‐bud stalks. These stalks were grown in an incubator at 28 ± 0.5°C 3000 lx, with a 16 h : 8 h, light : dark photoperiod, until the buds sprouted and the seedlings emerged, then three biological replicates were injected with *S. scitamineum* at 5 × 10^6^ spores ml^−1^ or water (the control) (Huang *et al*., 2018), respectively.

For testing the biological relevance of the 25‐amino‐acid peptide of the C‐terminal of SsPele1 (SsPel25) in sugarcane‐*S*. *scitamineum* pathosystem, three biological replicates each containing green sheaths from 10 ROC22 plants were surface‐sterilized in 3% NaClO (v/v, contained 0.02% Tween20) for *c*. 20 min and washed with sterilized water. The outermost leaf sheath was discarded and 40 inner leaf sheaths were cut into 80 slices at about 0.5 cm × 2 cm. These slices were divided into two equal parts and immersed into the smut fungus solution (diploid‐type Ss17‐18 at OD_600_ = 0.008 in water plus 0.02% Tween20) only or containing the SsPel25 peptide (5 μM), respectively, and then vacuumized (0.4 kg cm^−2^) for 10 min. They were placed on the filter paper covering solid Murashige & Skoog medium, and grown in an incubator (28 + 0.5°C, 3000 lx, 16 h : 8 h, light : dark). About 10 slices were pooling‐sampled at 0, 12 and 24 h for DNA isolation, respectively.

*Arabidopsis thaliana* Col‐0 was used for sugarcane and *S. scitamineum* gene transformation and protoplast isolation. Seeds were germinated in soil, and plants were grown in an incubator at 22°C, 60% relative humidity with a 16 h : 8 h, light : dark photoperiod.

### RNA isolation, cDNA amplification and quantitative real‐time (qRT)‐PCR

The sugarcane buds and Arabidopsis leaves were collected for RNA isolation using a TRIzol kit (#10296028; Invitrogen). RNA samples from NCo376 and YC71‐374 sugarcane genotypes infected with *S. scitamineum* at 0 d postinoculation (dpi), 3 dpi (Peters *et al*., 2017), 5 dpi (Schaker *et al*., 2016) and 7 dpi (Singh *et al*., 2004) were used for microarray hybridization and qRT‐PCR validation. Total RNA (1 μg) was used for cDNA synthesis and qRT‐PCR using ChamQ™ Universal SYBR qPCR Master Mix (#Q331‐02; Vazyme, Nanjing, China) on a QuantStudio 3 machine (Applied Biosystems, Foster City, CA, USA). In qRT‐PCR analysis, the relative expression level of sugarcane genes was normalized to the reference genes *acyl‐CoA dehydrogenase* and *serine/arginine repetitive matrix protein 1* (Livak & Schmittgen, 2001; Huang *et al*., 2018), and that of *S. scitamineum* genes was normalized to the reference genes *inosine 5′‐monophosphate dehydrogenase* and *SEC65‐signal recognition particle subunit* (Livak & Schmittgen, 2001; Huang *et al*., 2018), whereas that of Arabidopsis genes was normalized to *Actin* with $2^{‐\Delta \Delta C_{q}}$ method (Livak & Schmittgen, 2001; Wang *et al*., 2017).

### TaqMan based detection of *S. scitamineum*

The fungal biomass of *S. scitamineum* in the infected sugarcane buds and sheath slices were quantified using a TaqMan‐based qPCR method as described by Su *et al*. (2013). The cycle amplification of *bE*, a *S. scitamineum* gene related to mating (Albert & Schenck, 1996), in DNA samples and the plasmid pMD19‐*bE* was compared (Su *et al*., 2013). The primer pairs and the probes are listed in the Supporting Information Table S1.

### Microarray design, hybridization, validation and data analysis

In order to identify the differentially expressed genes (DEGs) during the *S. scitamineum*–sugarcane interaction, a customized 8 × 60 K Agilent microarray (Agilent Technologies, Santa Clara, CA, USA), targeting nonredundant sugarcane genes (20 392, Table S2) (Que *et al*., 2014a) and *S. scitamineum* genes (6621 coding sequences) (Que *et al*., 2014a), was designed. The probes against the sugarcane genes had two technical replicates, whereas those against the *S. scitamineum* genes had three technical replicates. The microarray hybridization and the systemic normalization of gene expression, general data analysis of sample groups and calculation of differential gene expression level were processed by Shanghai Biotechnology Co. Ltd (SBC, Shanghai, China). R software was used for normalization per chip and systemic normalization of sugarcane and smut gene differential expression level with the Quantile algorithm separately. The DEGs with expression fold‐change ≥ 2 or ≤ 0.5 (*P* < 0.05) were identified using the limma package (www.bioconductor.org) (Bolstad *et al*., 2003). All of these data were deposited in the Gene Expression Omnibus (GEO, GSE140801). Data analysis and figure illustration were performed using toolkit TBtools (Chen *et al*., 2020). For identifying the effector genes, the coding DNA sequences (CDSs) of *S. scitamineum* genes were aligned to the effector sequences of other gramineous smut fungi (Laurie *et al*., 2012; Ali *et al*., 2014; Brefort *et al*., 2014; Que *et al*., 2014a; Dutheil *et al*., 2016), using TBtools (Chen *et al*., 2020).

### Construction of a sugarcane–*S. scitamineum* co‐expression network and screening of key genes

The R package based wgcna (http://labs.genetics.ucla.edu/horvath/CoexpressionNetwork/Rpackages/WGCNA/) was used for identifying the DEG matrix (module) that was significantly associated with incubation time and fungal biomass. The parameters (soft threshold, 20; minimum module size, 30; merge cut height, 0.20) were fitted, and the modules, including DEGs with similar expression tendencies, were generated statistically and equitably with a one‐step automatic construction method and indicated by different colors according to the correlation patterns. The DEGs from those modules significantly associated with the increase of the fungal biomass and incubation period were chosen. The connectivity value between genes was obtained from the wgcna. The top DEGs with the highest connectivity value were used to generate the visible co‐expression network in Cytoscape (v.3.5.0) software (Shannon *et al*., 2002; Cline *et al*., 2007).

### Gene cloning, plasmid construction and sequence analysis

Sugarcane *PLANT ELICITOR PEPTIDE RECEPTOR1* (*ScPEPR1*) and four *S. scitamineum* putative effectors, *SsPE1*, *SsPE4*, *SsPE14* (*SsPele1*) and *SsPE15*, were cloned from genotype ROC22 (Table S3). The open reading frames (ORFs) of these genes were cloned into destination vectors, including pGBKT7, pGADT7, pSUC2T7M13ori, pFastR06, pXCSG and pCMABIA1306 (Table S1), using a cloning Kit (#C112‐01; Vazyme). Likewise, the ORFs of ScPEPR1 and SsPE14 were introduced into pCMABIA1300S‐nYFP and pCMABIA2300S‐cYFP, respectively, to generate N‐terminal YFP (nYFP) fused to ScPEPR1 by its N‐terminus (nYFP‐ScPEPR1) and C‐terminal (cYFP) fused to SsPE14 or SsPE14‐Δsp (without signal peptide) by its C‐terminus (SsPE14‐cYFP and SsPE14‐Δsp‐cYFP) (YFP, yellow fluorescent protein). The ORF of *AtPEPR1* without a stop codon also was introduced into pCAMBIA1306‐FLAG. The primer pairs used for plasmid construction are listed in Table S1.

The sequences of the PEPR and peptide1 (Pep1) were downloaded from Sequence Read Archive database (SRP192749) and NCBI database (*S. spontaneum* genomic data: GCA_003544955.1; Table S3). The alignment of PEPR, Pep1 and the effectors was performed with Dnaman (v.7.0.2.176) and Genedoc (http://www.flu.org.cn/en/download‐47.html). A phylogenetic tree was generated using the maximum‐likelihood method with 1000 bootstrap replicates in Mega7 software (Institute for Genomics and Evolutionary Medicine, Temple University, Philadelphia, PA, USA).

### Yeast two‐hybrid (Y2H) and glutathione S‐transferase (GST)‐pulldown experiments

For validating the protein interactions, prey and bait vectors containing genes as indicated in the figures were co‐transformed into Y‐2‐HGold chemically competent cells. Positive yeast clones containing two plasmids were selected from SD/‐Trp‐Leu medium (#630494; Clontech, Terra Bella Avenue Mountain View, CA, USA) and were re‐plated on SD/‐Trp‐Leu and SD/‐Trp‐Leu‐His‐Ade medium (#630494; Clontech).

After codon optimization and synthesis, the CDSs of *ScPEPR1‐N* and *SsPE14* were ligated into plasmids, generating pCzn1_*ScPEPR1*‐N and pGEX‐4T‐1_*SsPE14*, which were transformed into *Escherichia coli* strain BL21. The histidine (HIS)‐ and GST‐tagged proteins were purified and the *in vitro* GST pulldown experiments were performed according to the method of Tarun & Sachs (1996).

### Secretory function assay of signal peptide in yeast

The assay was performed mainly based on Xu *et al*. (2019). The sequence of the signal peptide was inserted into the vector pSUC2T7M13ori and transformed into yeast strain YTK121 (Jacobs *et al*., 1997). The transformed yeast was plated on the CMD‐W (minus tryptophane plates) (Xu *et al*., 2019), and incubated at 30°C for 3 d in darkness. For invertase secretion assay, transformers were replica plated on YPRAA plates (1% yeast extract, 2% peptone, 2% raffinose and 2 μg ml^−1^ antimicyn A) lacking glucose. The activity of invertase also was determined by reducing 2,3,5‐triphenyltetrazolium chloride (TTC) to insoluble red 1,3,5‐triphenylmethyl nitrogen (TPF) (Xu *et al*., 2019).

### Confocal images, protein extraction, immunoprecipitation and immunoblotting assay

The transformed *Agrobacterium tumefaciens* (GV3101) cells with plasmids pFastR06‐SsPele1‐eGFP, pXCSG‐SsPele1‐mYFP, pXCSG‐SsPele1‐∆C‐mYFP, pCMABIA1306‐ScPEPR1‐Flag, pCMABIA1306‐ZmPEPR1‐Flag, pXCSG‐UmPele1‐mYFP, pCMABIA1306‐ScPEPR1‐N‐Flag, pCMABIA1300S‐ScPEPR1‐nYFP, pCMABIA2300S‐SsPE14‐cYFP, or pCMABIA2300S‐SsPE14‐Δsp*‐*cYFP were grown at 28°C/200 rpm in lysogeny broth medium supplemented with kanamycin/spectinomycin (50 μg ml^−1^) and rifampicin (35 μg ml^−1^). Agrobacterial cells were collected and re‐suspended in MS salt buffer (MS‐salt, plus 200 mM acetosyringone) and injected into *Nicotiana benthamiana* leaves. Confocal images of the fluorescent signal in the *N. benthamiana* leaves were pictured on a laser confocal microscope Leica TCS SP8 (Leica, Wetzlar, Germany) after 48 h of agroinfiltration.

The *N. benthamiana* leaves were collected 48 h postagroinfiltration, ground in liquid nitrogen and lysed in extraction buffer EXB (50 mM Tris pH7.5, 150 mM NaCl, 10% (v/v) glycerol, 2 mM EDTA, 5 mM DTT, protease inhibitor (Roche), 0.1% Triton). Lysates were centrifuged for 15 min at 20 000 ***g*** at 4°C. Aliquots of supernatants were used as input samples. Immunoprecipitations (IPs) were conducted by incubating supernatants with 15 μl GFP‐Trap beads (#gta‐10; ChromoTek, Planegg‐Martinsried, Germany) in 1.5 ml tubes for 2 h at 4°C. Beads then were collected by centrifugation at 1000 ***g*** and washed four times with extraction buffer. Beads then were heated in 2×Laemmli loading buffer, and the proteins were separated by SDS‐PAGE and analyzed by immunoblotting. The antibodies used included anti‐GFP (#HT801; Transgen, Beijing, China), anti‐HA (#11867423001; Roche), anti‐FLAG (#ab1162; Abcam, Cambridge, UK), anti‐His (#ab15149; Abcam) and anti‐GST (#ab19256; Abcam).

### Modified immunoprecipitation for detecting the associations of peptides with ScPEPR1

After 48 h of transient expression in *N. benthamiana* leaves, ScPEPR1‐FLAG was extracted in extraction buffer EXB (50 mM Tris pH7.5, 150 mM NaCl, 10% glycerol, 2 mM ETDA, 0.1% Triton X‐100, 0.5% DTT and 1% protein inhibitors cocktail) and purified by incubating the supernatants with 15 μl anti‐FLAG agarose (#A4596; Sigma) in 1.5 ml tubes for 1 h at 4°C. After centrifugation at 1000 ***g***, the agarose gels were washed four times in extraction buffer and then incubated in 1 μg horseradish peroxidase (HRP)‐conjugated anti‐His antibodies (#ab1187; Abcam) and 10 mM his‐tagged peptides (his‐ScPep1, his‐SsPel25, or both of his‐ScPep1 and SsPel25) for 1 h at 4°C. The agarose gels then were washed three times in extraction buffer and transferred into a 96‐well plate for detecting HRP activity using chemiluminescence substrate (#37069; ThermoFisher, Waltham, MA, USA).

### Mitogen‐activated protein kinase (MAPK) assays on sugarcane, maize and Arabidopsis

For MAPK activation assays, 1 μM ScPep1 or ZmPep1 with or without 5 μM SsPel25 was added into sugarcane (Wang *et al*., 2020), maize (Cao *et al*., 2014) and Arabidopsis (Yoo *et al*., 2007) protoplasts, respectively. Then 1 μM AtPep1 peptide plus 0.02% silwet‐77 was sprayed onto the leaves of 4‐wk‐old Arabidopsis plants. Total protein samples were collected at the indicated time points and used for immunoblotting with anti‐p44/42 MAPK antibody (#4370; Cell Signaling, Danvers, MA, USA) to detect phosphorylation of MAPKs (Suarez‐Rodriguez *et al*., 2007).

### Transient expression and reporter assay in protoplasts

The mesophyll tissues of 4‐wk‐old Arabidopsis col‐0 plants were used for protoplast isolation and the transfection with DNA plasmids were performed according to Yoo *et al*. (2007). After that, protoplasts were incubated at room temperature under weak light for 16 h, and then used for protein immune‐binding and luciferase assays.

Protoplasts isolated from 4‐wk‐old Arabidopsis plants were co‐transfected with *proFRK1‐LUC* along with the indicated constructs as described (Li *et al*., 2005). At 16 h after transfection, the protoplasts were treated with 1 μM peptides as indicated. The luciferase (LUC) activity was determined at 2 h after the peptide treatments using the luciferase reporter system (#E1500; Promega).

### Generation of transgenic *A. thaliana* plants and powdery mildew infection

The *A. tumefaciens* cells (GV3101) containing plasmid pCAMBIA1306_ScPEPR1‐FLAG or pCAMBIA1301_SsPE14 were used for floral‐dipping to generate the transgenic Arabidopsis lines. For powdery mildew infection, the spores of *Golovinomyces cichoracearum* were blown onto the leaves of the *ScPEPR1*‐ and *SsPE14*‐overexpression lines. At 0, 3, and 5 dpi, leaves were collected for RNA isolation. The leaves at 5 dpi were collected for visualizing fungal structures using trypan blue staining (Frye & Innes, 1998).

### Statistical analysis

Statistical analysis of the qRT‐PCR data from three biological replicates was done by Spss Statistics (v.22.0.0.0; IBM, Armonk, NY, USA). Using two‐tailed Student’s *t*‐tests, SEs were calculated using the variance and covariance values obtained from the linear model fitting. The expression level was shown as the mean ± SD.

## Results

### Gene co‐expression network during sugarcane–*S*. *scitamineum* interaction

**Fig. 1:**
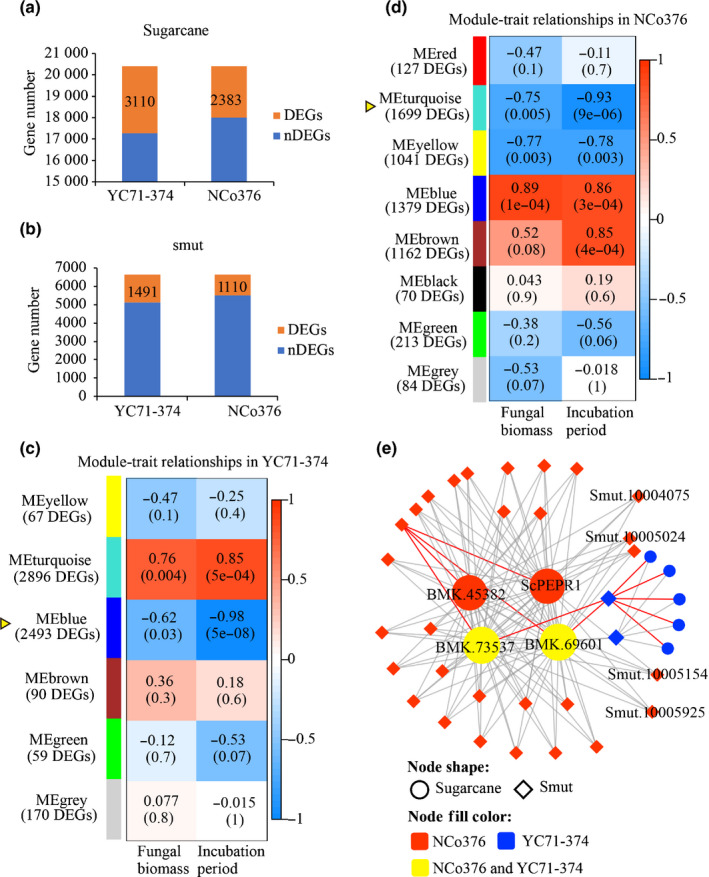
Gene co‐expression network in sugarcane–*Sporisorium scitamineum* interaction. (a) The total numbers of differentially expressed sugarcane genes in sugarcane susceptible genotype YC71‐374 and resistant genotype NCo376 at 3, 5, 7 d postinfection with *S. scitamineum*. DEG, differentially expressed gene; nDEG, not‐differentially expressed gene. (b) The total numbers of *S. scitamineum* DEGs at 3, 5, 7 d postinfection in sugarcane YC71‐374 and NCo376. (c, d) Module (DEG matrix)‐trait associated analysis bases on the correlation of the fungal biomass of *S. scitamineum* and incubation period with the expression level of the DEGs (from both sugarcane and smut) in YC71‐374 (c) and in NCo376 (d). The table exhibits modules in rows and traits in columns (fungal biomass and incubation period). The corresponding correlation value *r* and *P*‐value (in closing bracket) between row and column was shown in the color cell. The cell color was correlated to *r*‐value indicated by the color legend on the right, while the color legend on the left represented the row name. ME, module eigengenes. (e) Weighted gene co‐expression network between the top sugarcane kinase DEGs and smut secreted protein genes. The four biggest dots in the network represent sugarcane *mitogen‐activated protein kinase kinase kinase A* (MAPK, BMK.45382), *mitogen‐activated protein kinase* (BMK.69601), *receptor‐like serine/threonine‐protein kinase* (BMK.73537) and *plant elicitor peptide receptor1* (*PEPR1*) gene. The red line indicates the positive correlation between genes, whereas the grey line indicates the negative correlation. The size of node reflects the number of connections.

We used a customized microarray targeting both the representative sugarcane genes and *S. scitamineum* genes to identify the DEGs in the host and the fungus along with the progress of the infections (0, 3, 5 and 7 dpi). The smut fungus grew faster and was more abundant in the susceptible sugarcane cultivar YC71‐374 than in the resistant NCo376 (Fig. S1a), confirming the successful *S. scitamineum* infection. There was a uniform gene expression distribution as revealed by a boxplot (Fig. S1b), and all biological replicates had a strong correlation and good repeatable performance (*R* ^2^ ≥ 0.90) (Fig. S1c). Subsequently, compared with the samples at 0 dpi, we identified 3110 sugarcane DEGs in YC71‐374 and 2383 DEGs in NCo376 under the smut infection (Fig. 1a). Meanwhile 1491 and 1110 smut DEGs, including 94 putative effector genes, were identified respectively, after inoculation on YC71‐374 and NCo376 (Fig. 1b). As shown in Fig. S2, the expression tendency of 13 DEGs in qRT‐PCR analysis was consistent with the microarray results, indicating the high reliability of the microarray data.

We then performed a WGCNA analysis on the microarray data to construct gene co‐expression network. It resulted in six and eight co‐expression modules in the susceptible YC71‐374 (Fig. 1c) and the resistant NCo376 (Fig. 1d), respectively. Notably, both the blue module in YC71‐374 and the turquoise module in NCo376 with the lowest *P*‐value (*P* = 5 × 10^–8^ and *P* = 9 × 10^–6^, respectively) were highly and negatively correlated with incubation period (*r* = −0.98 and *r* = −0.93 respectively) and fungal biomass (*r* = −0.62, *P* = 0.03 and *r* = −0.75, *P* = 0.005, respectively) (Fig. 1c,d). We speculated that sugarcane genes in these two modules were likely involved in defense responses, and that their expression was suppressed by *S. scitamineum*. Then, 2959 sugarcane genes and 751 *S. scitamineum* genes from these two modules were grouped together to calculate the connectivity value between genes. The top sugarcane kinase genes and smut effector genes with the highest connectivity value subsequently were selected to construct the gene co‐expression network, where the genes with a WGCNA edge weight > 0.15 were connected with lines (Fig. 1e). The four biggest dots represented sugarcane genes: *mitogen‐activated protein kinase kinase kinase A* (BMK.45382), *mitogen‐activated protein kinase* (BMK.69601), *receptor‐like serine/threonine‐protein kinase* (BMK.73537) and *plant elicitor peptide receptor1* (*ScPEPR1*, BMK.75743). As PEPR1‐signaling is an important component in plant immune system (Tang & Zhou, 2016), we focused on the *ScPEPR1* gene for further analysis here (studies on other three genes will be reported elsewhere). The expression of *ScPEPR1* was decreased in either NCo376 or YC71‐374 after *S. scitamineum* infection, which accorded with the results of our microarray data (Fig. S2).

### ScPEPR1 is a phylogenetical ortholog to Arabidopsis PEPR1

The protein sequence analysis showed that ScPEPR1 contains N‐terminal extracellular LRR domain (a LRRNT_2, a LRR1 and a LRR8), a transmembrane domain and a C‐terminal cytoplasmic kinase domain, sharing the closest relationship to PEPR1s from *Sorghum bicolor* and *Zea mays* (Fig. S3a). The alignment of protein sequence showed that PEPR1 and its ortholog protein share 68.78% identity in amino acid sequence (Fig. S3b). And we confirmed that as a PRR receptor, YFP fused ScPEPR1 had a membrane localization in the leaf cells when transiently expressed in *N. benthamiana* (Fig. S3c).

We expressed ScPEPR1 under the control of the constitutive 35S promoter (*35S‐ScPEPR1*) in Arabidopsis. Compared to the expression of endogenous *AtPEPR1* in the control transgenic line (transformed with empty vector), *ScPEPR1* were highly expressed in the *35S‐ScPEPR1* lines (Fig. S4a). We found that the *35S‐ScPEPR1* lines showed fewer fungi and significantly fewer conidiophores on the leaves infected with powdery mildew *G. cichoracearum* at 5 dpi than the control line (Fig. S4b,c). The fungal‐induced expression of the defense‐related gene *AtWRKY33* (Gravino *et al*., 2017) (Fig. S4d) and the SA‐induced gene *AtPR5* (Sun *et al*., 2018) (Fig. S4e) were higher in *35S‐ScPEPR1* lines than in the control line at 3 and 5 dpi. We concluded that overexpression of the sugarcane *ScPEPR1* gene in Arabidopsis enhances plant resistance to powdery mildew. Thus, *ScPEPR1* gene is a structural, phylogenetic orthologous to Arabidopsis *PEPR1* and is functional in plant immunity.

### ScPEPR1 interacts with *S. scitamineum* effector SsPE14

**Fig. 2:**
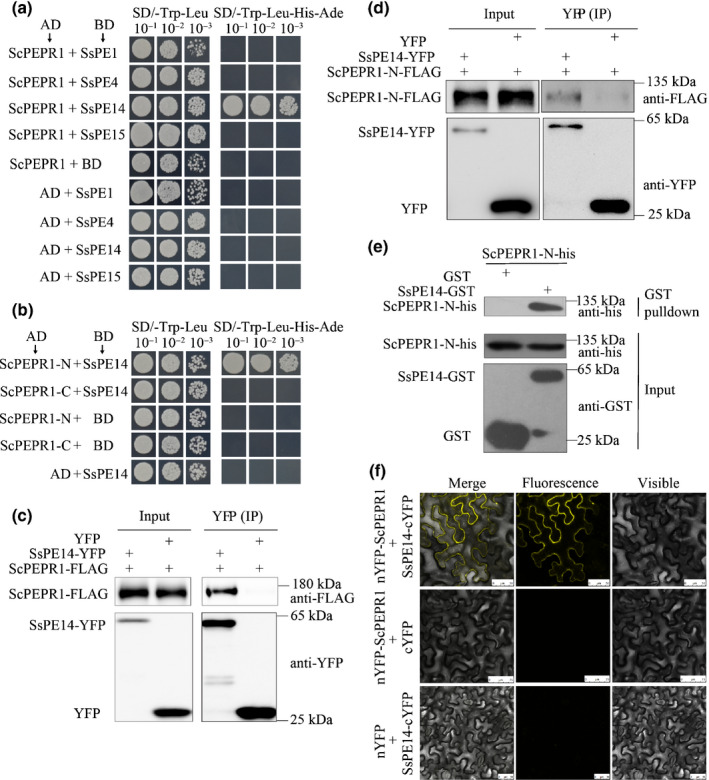
Sugarcane PLANT ELICITOR PEPTIDE RECEPTOR1 (ScPEPR1) interacts with the smut effector SsPE14. (a) ScPEPR1 interacts with SsPE14 in yeast. The activation domain (AD) and DNA binding domain (BD) plasmids containing the indicated genes were co‐transformed into yeast strain Y‐2‐Hgold and screened on synthetic dextrose dropout (SD) media lacking Leu and Trp (SD/‐Leu‐Trp). The single colonies were serially diluted and spotted onto SD/‐Leu‐Trp and SD/‐Leu‐Trp‐His‐Ade to observe the yeast cell growth. Yeast co‐transformed with AD‐largeT + BD‐p53 or AD‐largeT + BD‐laminC served as a positive control and negative control, respectively. EV, empty vector. SsPE1/4/14/15, *Sporisorium scitamineum* putative effector1/4/14/15. (b) The interaction between ScPEPR1 and SsPE14 in *Nicotiana benthamiana*, revealed by bimolecular fluorescence complementation assay. The *N. benthamiana* leaves were co‐infiltrated with *35S:nYFP‐ScPEPR1* and *35S:SsPE14‐cYFP*. Images were captured by a confocal microscope 2 d after *Agrobacterium tumefaciens* transformation. Bar, 10 μm. (c) Co‐immunoprecipitation (co‐IP) analysis of interactions between ScPEPR1‐FLAG and SsPE14‐YFP in *N. benthamiana* leaves. Proteins in total extracts (Input) and after IP with GFP‐trap beads (IP (YFP)) were detected on immunoblots using α‐FLAG or α‐GFP antibodies. GFP/YFP, green/yellow fluorescent protein. (d) SsPE14 interacts with N‐terminal LRR domain of ScPEPR1 (ScPEPR1‐N), but not with the C‐terminal of ScPEPR1 (ScPEPR1‐C) in yeast. The experiment was performed according to the same procedure in (a). (e) SsPE14 interacts with the ScPEPR1‐N, revealed using glutathione S‐transferase (GST) pull‐down assays. The recombinant ScPEPR1‐N‐his and SsPE14‐GST proteins purified from *Escherichia coli* BL21 strain were subjected to a GST pull‐down analysis. Proteins in input and pull‐down were detected on immunoblots using α‐his or α‐GST antibodies. (f) Co‐IP analysis of interactions between SsPE14‐YFP and ScPEPR1‐N‐FLAG in *N. benthamiana* leaves. Proteins in total extracts (Input) and after IP with GFP‐trap beads (IP (YFP)) were detected on immunoblots using α‐FLAG or α‐GFP antibodies. These experiments were repeated at least three times with similar results.

We then tested the possible interactions between ScPEPR1 and the *S. scitamineum* putative effectors (SsPEs) in the WGCNA co‐expression network (Fig. 1e; Table S4). In the Y2H assay, ScPEPR1 specifically interacted with SsPE14 (smut.10005024) (Fig. 2a). In the following protein truncation tests, we found that the N‐terminal extracellular LRR domain (ScPEPR1‐N) but not the C‐terminal cytoplasmic kinase domain (ScPEPR1‐C) of ScPEPR1 interacted with SsPE14 (Fig. 2b).

In order to further examine the protein interactions *in vivo*, co‐immunoprecipitation (co‐IP) assays were performed after transient expression of YFP‐tagged SsPE14 and FLAG‐tagged ScPEPR1 or ScPEPR1‐N in *N*. *benthamiana*. As shown in Fig. 2(c,d), ScPEPR1‐FLAG or ScPEPR1‐N‐FLAG was co‐purified with SsPE14‐YFP, rather than with the YFP control. Furthermore, in an *in vitro* pull‐down assay, the direct interaction between SsPE14‐GST and ScPEPR1‐N‐His also was observed (Fig. 2e). Together, these results demonstrated that SsPE14 interacts with ScPEPR1 by its N‐terminal LRR domain.

As SsPE14 interacts with extracellular LRR domain of ScPEPR1, we intended to visualize the subcellular location of the protein complexes formed by the two proteins using bimolecular fluorescence complementation (BiFC) assay. For this, N‐terminal YFP (nYFP) was fused to ScPEPR1 by its N‐terminus (nYFP‐ScPEPR1) and C‐terminal YFP (cYFP) was fused to SsPE14 by its C‐terminus (SsPE14‐cYFP). Yellow fluorescence was observed on the cytoplasmic membrane of the leaf cells upon transient co‐expression of nYFP‐ScPEPR1 and SsPE14‐cYFP in *N*. *benthamiana*, suggesting that ectopically expressed SsPE14 could be localized to the apoplastic space, where it interacts with extracellular LRR domain of ScPEPR1 (Fig. 2f).

### SsPE14 is a plant elicitor peptide‐like effector

**Fig. 3:**
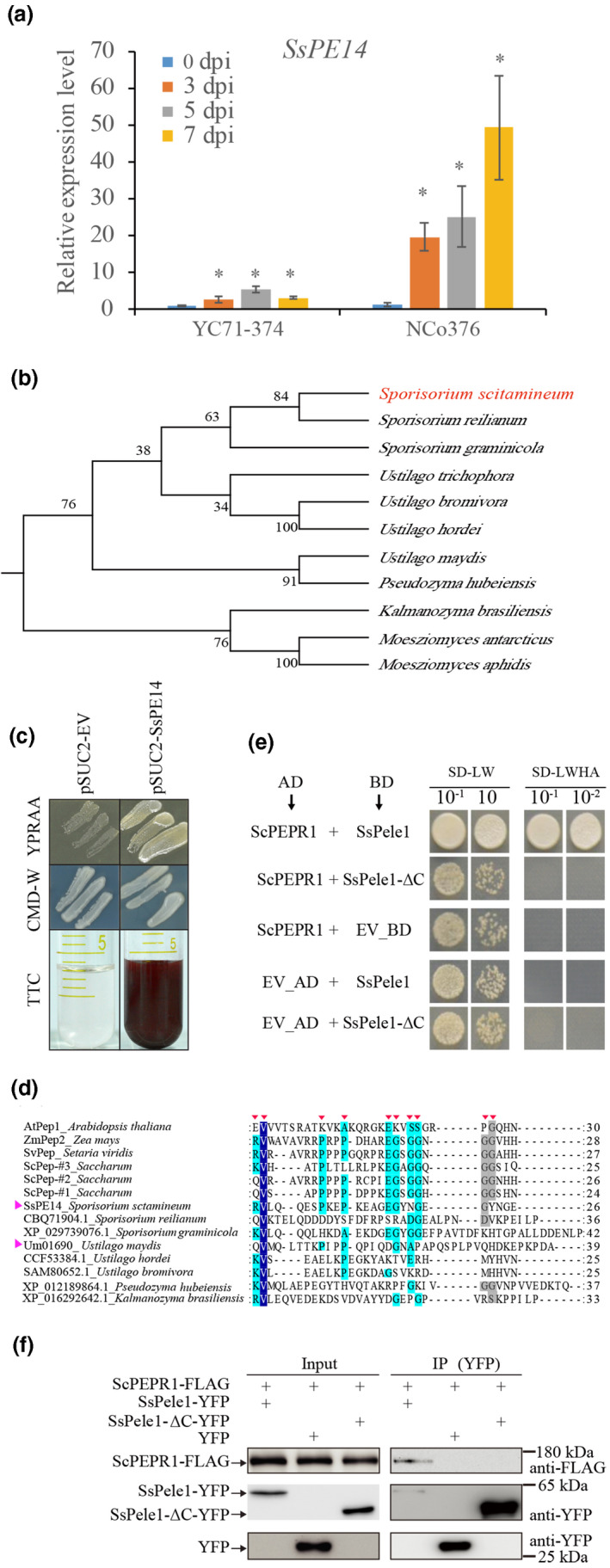
The characterization of the smut fungal effector SsPE14. (a) Quantitative real‐time PCR analysis of expression of *Sporisorium scitamineum* effector *SsPE14* in sugarcane genotypes YC71‐374 and NCo376 at indicated time points. The bars indicate relative fold‐change ± SD (*n* = 3) compared to 0 dpi. *indicates a significant difference between the 0 dpi, and 3, 5 or 7 dpi (*P* < 0.05) determined by Student’s *t*‐test. dpi, d postinoculation. (b) Phylogenetic analysis of SsPE14 and its orthologs from *Sporisorium graminicola*, *Sporisorium reilianum*, *Ustilago maydis*, *Ustilago trichophora*, *Ustilago bromivora*, *Ustilago hordei*, *Pseudozyma hubeiensis*, *Moesziomyces antarcticus*, *Moesziomyces aphidis* and *Kalmanozyma brasiliensis*; Numbers at the branches indicate bootstrap values. The accession number of the sequences used in the present study can be found in Supporting Information Table S3. (c) Experimental validation of the signal peptide of SsPE14 using the yeast invertase secretion assay. Yeast YTK121 strains carrying the SsPele1 fused in frame to the invertase gene in the pSUC2 vector can grow in the CMD‐W media (with sucrose) and YPRAA media (with raffinose instead of sucrose, growth only when invertase is secreted). The secreted invertase also can reduce triphenyltetrazolium chloride (TTC) to red formazan. The negative control was YTK121 strains carrying the pSUC2 vector. (d) The alignment of the conserved amino acid sequences of plant elicitor peptides (Arabidopsis AtPep1, maize ZmPep2, green foxtail SvPep and sugarcane ScPep‐#1/#2/#3) and the fungal homologs of SsPele1 (from *S. reilianum*, *S. graminicola*, *U. maydis*, *U. hordei*, *U. bromivora*, *P. hubeiensis*, and *K. brasiliensis*) obtained from the NCBI database (Table S3). The highly conserved sites are colored and marked with red triangle symbol on the top row. SsPele1 and UmPele1 (Um01690) marked with purple triangle symbol. The accession number of the sequences used in the present study can be found in Table S3. (e) ScPEPR1 interacts with SsPele1 and SsPele1‐ΔC in yeast. SsPele1‐ΔC is the fragment of SsPele1 with deletion of 68 residues in its C‐terminus. The activation domain (AD) and binding domain (BD) plasmids containing the indicated genes were co‐transformed into yeast strain Y‐2‐Hgold and screened on synthetic dextrose dropout media lacking Leu and Trp (SD/‐Leu‐Trp). The single colonies were serially diluted and spotted onto SD/‐Leu‐Trp and SD/‐Leu‐Trp‐His‐Ade to observe the yeast cell growth. Yeast co‐transformed with AD‐largeT + BD‐p53 or AD‐largeT + BD‐laminC served as a positive control and negative control, respectively. EV, empty vector. Three independent experiments gave consistent results. Pele1, plant Pep1‐like effector 1. (f) Co‐immunoprecipitation (co‐IP) analysis of interactions between ScPEPR1‐FLAG and SsPele1‐ΔC‐YFP in *Nicotiana benthamiana* leaves. Proteins in total extracts (Input) and after IP with GFP‐trap beads (IP (YFP)) were detected on immunoblots using α‐FLAG or α‐GFP antibodies. These experiments were repeated at least three times with similar results. GFP/YFP, green/yellow fluorescent protein.

The expression of *SsPE14* is strongly induced during smut infection (Fig. 3a), suggesting that it might play an important role in promoting virulence of *S*. *scitamineum*. In the NCBI database, two orthologs of SsPE14 in *Sporisorium* species, four in *Ustilago* species, one in *Pseudozyma* species and two in *Moesziomyces* species were found through Blastp tool. Phylogenetic tree analysis showed that SsPE14 was closely related to two orthologs from *Sporisorium* (Fig. 3b). SsPE14 is likely a secreted protein without a conserved domain in the NCBI database (https://www.ncbi.nlm.nih.gov/Structure/cdd/wrpsb.cgi) (Que *et al*., 2014a). It also is predicted to be an effector protein (Ratio 0.739) by EffectorP (http://effectorp.csiro.au/). Thus, SsPE14 might be a conserved effector protein in smut fungi species.

SsPE14 has a predicted signal peptide on its N‐terminus. To functionally validate it, we used a genetic assay based on the requirement of invertase secretion for yeast cells to grow on media containing raffinose as sole carbon source. The predicted signal peptide sequence of *SsPE14* was fused in‐frame to the yeast invertase gene in the vector pSUC2T7M13ori (Xu *et al*., 2019). The invertase mutant yeast strain YTK121 transformed with pSUC2T7M13ori‐*SsPE14* construct grew on the YPRAA medium (sucrose was replaced by raffinose, the YTK121 can grow only when invertase is secreted) (Fig. 3c). The invertase secretion was further confirmed using an enzymatic activity assay based on invertase‐mediated conversion of the colorless dye TTC into the insoluble red colored triphenylformazan (Fig. 3c). These results demonstrated that the signal peptide of SsPE14 is functional and SsPE14 is a secreted protein.

Next, a BiFC experiment was performed to test interaction between nYFP‐ScPEPR1 and SsPE14‐∆sp‐cYFP whose secretion signal peptide has been deleted. We found that SsPE14‐∆sp‐cYFP did not interact with nYFP‐ScPEPR1 (Fig. S5). This result indicates that SsPE14 must be secreted into apoplastic space for interaction with ScPEPR1 LRR domain.

### The C‐terminal Pep1 like domain is required for SsPele1 to interact with ScPEPR1

The interaction between the SsPE14 and the extracellular LRR domain of ScPEPR1 (Fig. 2d–f) is reminiscent of the perception of Arabidopsis AtPep1 by AtPEPR1 (Yamaguchi *et al*., 2006; Tang *et al*., 2015). We wondered whether there was a sequence similarity between SsPE14 and plant elicitor peptides. Hence, the conserved amino acid residues from the plant elicitor peptides and fungal orthologs of SsPE14 were aligned (Figs 3d, S6). We found that the C‐terminal 26 amino acids of SsPE14 (149–174 aa) and its orthologs had several conserved sites compared with the plant elicitor peptides (Peps) (Fig. 3d). Hereafter, the SsPE14 was renamed as SsPele1 (*S. scitamineum* plant elicitor peptide‐like effector 1).

We then tested whether SsPele1 interacts with ScPEPR1 through its C‐terminal Pep1 like domain using Y‐2‐H and Co‐IP assays. We found that deletion of Pep1 like motif (SsPele1‐∆C) in SsPele1 abolished its association with ScPEPR1 in Y‐2‐H experiments (Fig. 3e). Consistently, SsPele1‐∆C‐YFP did not interact with ScPEPR1‐FLAG in Co‐IP experiments (Fig. 3f). These data demonstrate that Pep1 like domain of SsPele1 is required for its association with ScPEPR1.

### ScPep1‐, but not SsPele1‐, perception by ScPEPR1 induces immune responses

Three orthologs of AtPep1 (NP_569001.1) in sugarcane were identified by protein blast against our RNA‐seq data (SRP192749) and the published genomic data (*S. spontaneum*, GCA_003544955.1). The sugarcane Peps, namely ScPep‐#1, ScPep‐#2 (ScPep1) and ScPep‐#3 (Table S3), were aligned with the plant elicitor peptides (AtPep1 from Arabidopsis, ZmPep2 from maize and SvPep from *Setaria viridis*, Table S3) to assess their similarity. Clearly, the sugarcane Peps contain the conserved amino acids in their C‐terminus compared with known plant Peps (Figs 3d, S6).

**Fig. 4:**
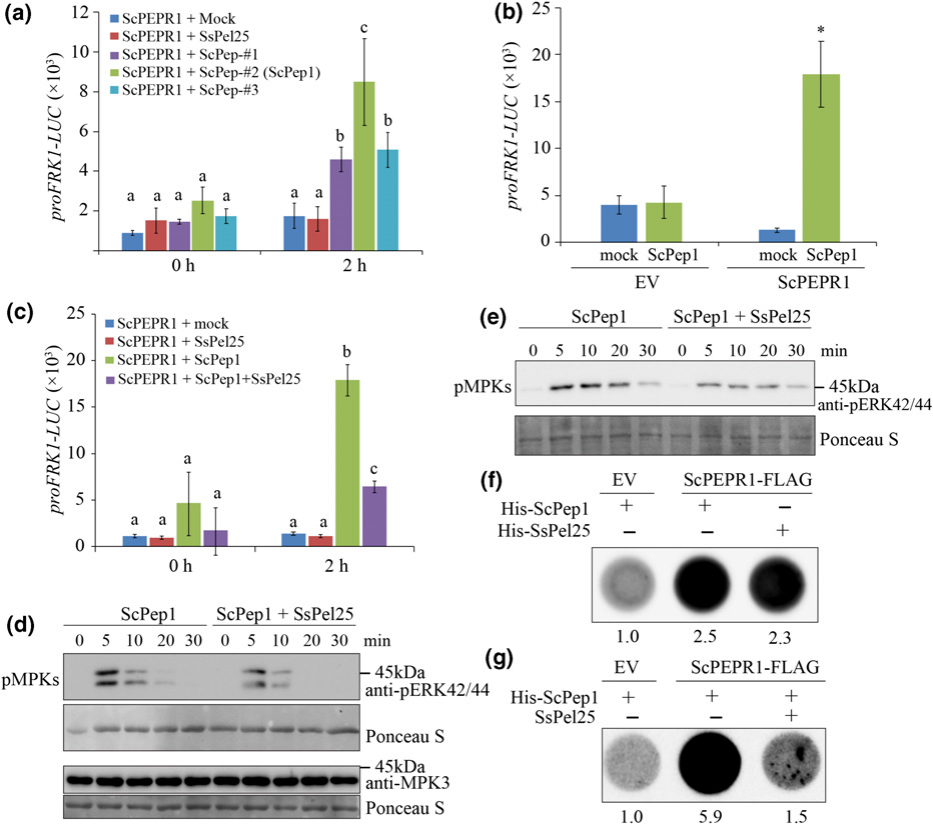
The smut effector SsPele1 suppresses sugarcane peptide1 (ScPep1)‐induced immune responses. (a) Sugarcane ScPep1 candidates induce *proFRK1‐LUC* expression in Arabidopsis protoplasts. The Arabidopsis ecotype Col‐0 protoplasts were transfected with *proFRK1‐LUC* along with *ScPEPR1*. Sixteen hours later, the protoplasts were treated with 0.2 μM ScPep1‐#1, #2, #3, or the 25‐amino‐acid peptide from SsPele1 (SsPel25), then the *LUC* reporter activity was determined 2 h later (LUC, luciferase). Error bars indicate the SD. Different letters indicate statistical significance (*P* ≤ 0.01) determined by one‐way ANOVA followed by Tukey’s honestly significant difference (HSD) tests. (b) ScPep1‐induced *proFRK1‐LUC* expression is dependent on sugarcane *PLANT ELICITOR PEPTIDE RECEPTOR1* (*ScPEPR1*). Col‐0 protoplasts were transfected with *proFRK1‐LUC* along with *ScPEPR1*or the empty vector (EV). Sixteen hours later, the protoplasts were treated with 0.2 μM ScPep1 for another 2 h, and the *LUC* reporter activity was determined. * indicate statistical significance (*P* ≤ 0.01) to the mock determined by one‐way ANOVA followed by Tukey’s HSD tests. (c) The SsPel25 peptide inhibits ScPep1‐induced *proFRK1‐LUC* expression. Col‐0 protoplasts were transfected with *proFRK1‐LUC* along with *ScPEPR1*. The assays were done as in (a). (d, e) SsPel25 suppresses ScPep1‐induced phosphorylation of MAPKs (pMPKs). Col‐0 (d) and sugarcane (e) protoplasts expressing *ScPEPR1* were treated with 1 μM ScPep1 or ScPep1 plus 2 μM SsPel25, and total protein extracts were prepared at the indicated time points. The phosphorylation of MAPKs was detected on an immunoblot probed with anti‐p44/42 MAPK antibody. (f, g) His tagged ScPep1 and SsPel25 peptides associate with ScPEPR1‐FLAG (f), and SsPel25 competes the interaction between His‐ScPep1 and ScPEPR1‐FLAG (g). The transient expression of ScPEPR1‐FLAG samples extracted and purified from *Nicotiana benthamiana* leaves with anti‐FLAG agarose, and then then incubated in 1 μg horseradish peroxidase (HRP)‐conjugated anti‐His antibodies (Abcam; #ab1187) and 10 mM his‐tagged peptides or no tagged peptides (his‐ScPep1, his‐SsPel25, or both of his‐ScPe1 and SsPel25). The agarose gels then were washed three times in extraction buffer and transferred into a 96‐well plate for detecting HRP activity using chemiluminescence substrate (ThermoFisher; #37069). The dot intensity indicates the interactions between ScPEPR1‐FLAG and His‐tagged peptides. These experiments were repeated at least three times with similar results.

In order to test activity of the putative sugarcane Pep1 candidates, we synthesized three peptides of ScPeps, ScPep‐#1, ScPep‐#2 and ScPep‐#3, and monitored their ability for induction of *proFRK1‐LUC* in an Arabidopsis protoplast, which is a transient reporter system widely used in studying PTI signaling (Asai *et al*., 2002). *FRK1* encodes a receptor‐like kinase that is rapidly induced by PAMPs (Asai *et al*., 2002). When co‐expressing with ScPEPR1, ScPeps, especially ScPep‐#2, strongly induced the expression of *proFRK1‐LUC* (Fig. 4a). Thus, we named it as ScPep1. ScPep1 could not induce *proFRK1‐LUC* expression in the absence of *ScPEPR1* (Fig. 4b), indicating that ScPep1 is specifically perceived by ScPERP1, but not by AtPEPR1 in Arabidopsis protoplasts. Strikingly, the 25‐amino‐acid‐peptide from SsPele1 C‐terminal Pep1 like domain (SsPel25) (Fig. 3d) did not induce *proFRK1‐LUC* reporter (Fig. 4a), despite its sequence similarity to plant Peps.

### SsPel25 inhibits ScPep1‐induced immune responses

The interaction of SsPele1 with ScPEPR1 (Fig. 2) without activating the ScPEPR1‐mediated *FRK1* expression (Fig. 4a) promoted us to test whether it suppresses ScPEPR1‐signaling. We found that the co‐application of SsPel25 peptide significantly suppressed the ScPep1‐induced expression of the *proFRK1‐LUC* reporter in the Arabidopsis protoplasts expressing ScPEPR1 (Fig. 4c). In the same protoplasts, we examined the ScPep1‐induced phosphorylation of MAPKs, an early event in PTI responses, with or without SsPel25. As shown in Fig. 4(d), the ScPep1‐induced transient phosphorylation of MAPKs was reduced in the presence of SsPel25. Furthermore, we examined whether SsPel25 inhibits ScPep1‐induced phosphorylation of MAPKs on sugarcane. Consistently, we found co‐application of SsPel25 significantly suppressed ScPep1‐induced phosphorylation of MAPK in sugarcane protoplasts (Fig. 4e). Moreover, application of SsPel25 promoted *S. scitamineum* propagation on sugarcane sheath tissue (Fig. S7). Together, these results indicated that SsPel25 inhibits ScPep1‐induced immune responses.

### SsPel25 competes with ScPep1 to bind to ScPEPR1

We then sought to examine whether SsPel25 competes with ScPep1 for perception by ScPEPR1. First, the FLAG‐tagged protein ScPEPR1‐FLAG or an empty vector as control was transiently expressed in *N. benthamiana* and total protein extracts were incubated with anti‐FLAG agarose. After washing, the anti‐FLAG agarose binding ScPEPR1‐FLAG were incubated with mock, the 6× his‐tagged peptides His‐ScPep1 or His‐SsPel25, respectively, plus anti‐His‐HRP antibody for detecting the presence of his‐tagged peptides in the immune‐complexes in 96‐well plates (details in the Materials and Methods section). Both His‐ScPep1 and His‐SsPel25 were detected in the ScPEPR1‐FLAG immune‐complexes (Fig. 4f), indicating that both peptides bound with ScPEPR1. To check for competition between ScPep1 and SsPel25 for binding to ScPEPR1, the ScPEPR1‐FLAG‐binded anti‐FLAG agarose was incubated with His‐ScPep1 in the absence or presence of un‐tagged SsPel25. As shown in Fig. 4(g), the presence of SsPel25 greatly reduced the signal of His‐ScPep1 in the immune‐complexes, suggesting that SsPel25 indeed competed the association between ScPep1 and ScPEPR1.

### Overexpression of *SsPele1* in Arabidopsis suppresses AtPep1‐induced immunity

**Fig. 5:**
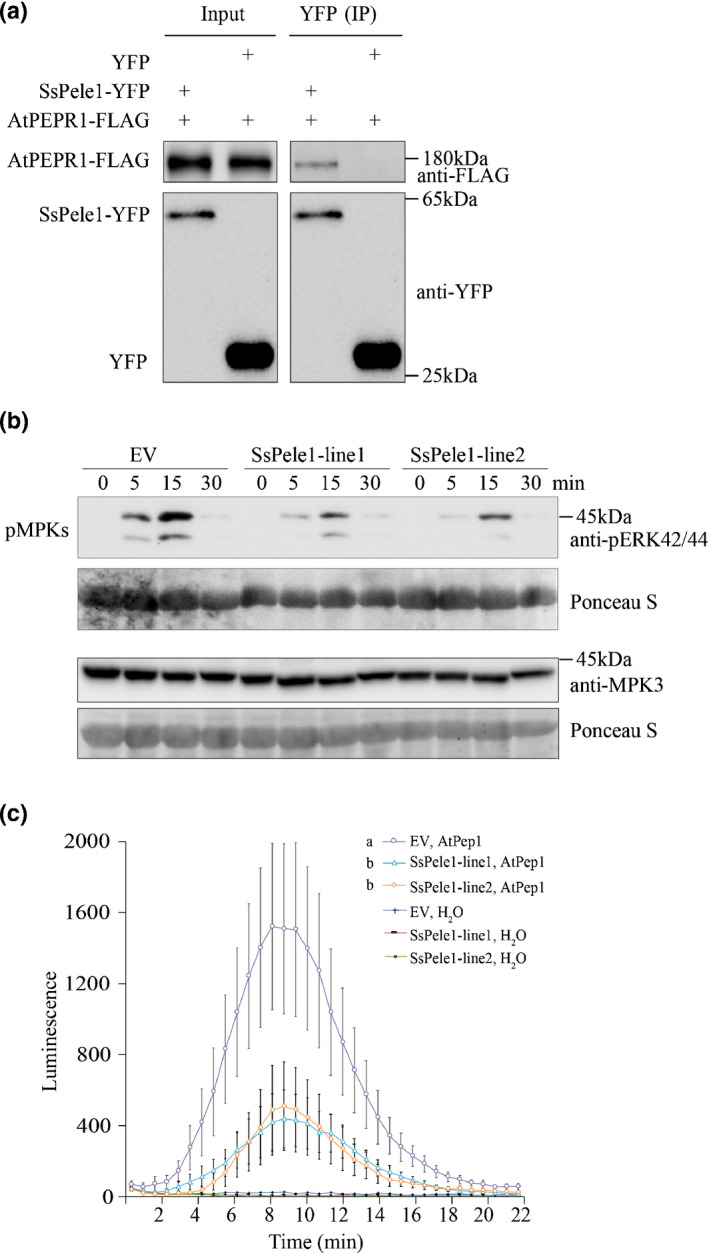
Overexpression of smut effector gene *SsPele1* in Arabidopsis suppresses peptide1 (AtPep1)‐induced immunity. (a) Co‐immunoprecipitation (co‐IP) analysis of the interactions between SsPele1‐YFP and AtPEPR1‐FLAG in *Nicotiana benthamiana*). Proteins in total extracts (Input) and after IP with GFP‐trap beads (IP (YFP)) were detected on immunoblots using anti‐FLAG or anti‐GFP antibodies (GFP/YFP, green/yellow fluorescent protein). (b) AtPep1‐induced phosphorylation of mitogen‐activated protein kinases (MAPKs) on *ScPele1* transgenic line1/2 and empty‐vector (EV) transgenic line. Total protein extracts were prepared from leaves of 4‐wk‐old transgenic Arabidopsis lines expressing *SsPele1* or an EV at the indicated time points after 1 μM AtPep1 treatment. The phosphorylation of MAPKs was detected on an immunoblot probed with anti‐p44/42 MAPK antibodies. Ponceau staining of the blot shows equal sample loading. (c) AtPep1‐induced H_2_O_2_ production was reduced in the *SsPele1* transgenic lines. The results shown are representative of three independent experiments. Each data point consists of six to eight replicates. Values are means ± SD. Different letters indicate statistical significance (*P* < 0.01) determined by one‐way ANOVA followed by Tukey’s honestly significant difference. These experiments were repeated three times with consistent results.

We generated transgenic Arabidopsis lines expressing *SsPele1* driven by 35S promoter (*35S‐SsPele1*). As AtPep1 is perceived by AtPEPR1 in Arabidopsis, we questioned whether SsPele1 interacts with and interferes with AtPEPR1 activation. Co‐IP experiments in *N. benthamiana* showed that SsPele1 also interacted with AtPEPR1 *in planta* (Fig. 5a). We then evaluated the plant immune responses to AtPep1 in the transgenic lines. First, AtPep1‐induced activation of MAPKs was examined. The leaves of 4‐wk‐old plants were treated with 1 μM AtPep1, and the total protein was collected at the indicated time points (Fig. 5b). The immunoblots with an anti‐pERK antibody showed that the AtPep1‐induced transient phosphorylation of MAPKs was reduced in the *35S‐SsPele1* lines than in the control line (Fig. 5b). We next examined the AtPep1‐induced production of ROS, another typical PTI response, in the *35S‐SsPele1* lines. As shown in Fig. 5(c), the AtPep1‐induced ROS were compromised in the *35S‐SsPele1* lines compared with the control line. Together, these results indicated that overexpression of *SsPele1* in Arabidopsis suppresses AtPep1‐induced immune responses.

### *Ustilago maydis* effector UmPele1 interacts with ZmPEPR1 and suppresses ZmPEPR1‐mediated immunity

**Fig. 6:**
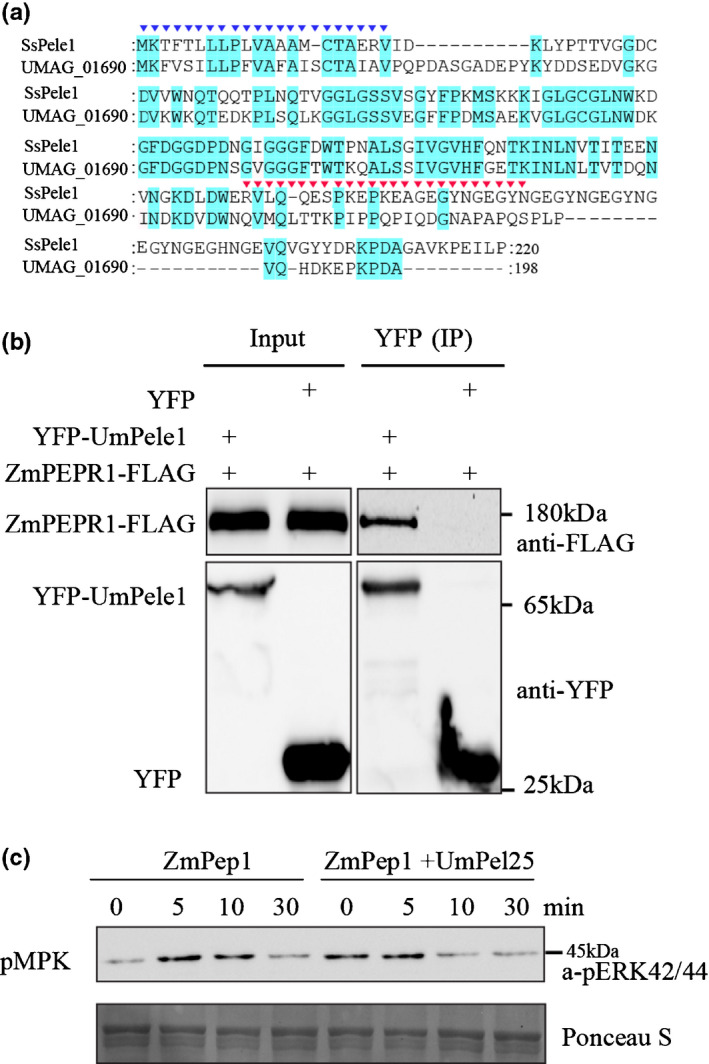
Smut effector SsPele1’s ortholog, UmPele1, interacts with maize PLANT ELICITOR PEPTIDE RECEPTOR1 (ZmPEPR1) and suppresses immune responses. (a) The alignment of *Ustilago maydis* ortholog (um01690, Supporting Information Table S3) and SsPele1. The N‐terminal amino acids representing the signal peptide and the C‐terminal conserved residues were marked in blue triangle symbol and red triangle symbol, respectively. The accession number of the sequences used in the present study can be found in Table S3. (b) Co‐immunoprecipitation (co‐IP) analysis of interactions between (*Zea mays*) ZmPEPR1‐FLAG and UmPele1‐YFP in *Nicotiana benthamiana* leaves. Proteins in total extracts (Input) and after IP with GFP‐trap beads (IP (YFP)) were detected on immunoblots using α‐FLAG or α‐GFP antibodies (GFP/YFP, green/yellow fluorescent protein). (c) UmPel25 suppresses ZmPep1 induced phosphorylation of mitogen‐activated protein kinases (MAPKs). Maize protoplasts expressing *ZmPEPR1* were treated with 1 μM ZmPep1 or 1 μM ZmPep1 plus 2 μM UmPel25, and total protein extracts were prepared at the indicated time points. The phosphorylation of MAPKs was detected on an immunoblot probed with anti‐p44/42 MAPK antibody. These experiments were repeated at least three times with similar results.

*Ustilago maydis* has an *SsPele1* ortholog effector gene, *Um01690* (Schilling *et al*., 2014) (Fig. 3d). The deletion of *Um01690* impaired fungal tumor induction on maize seedling leaves (Schilling *et al*., 2014), indicating Um01690 is an important effector for fungal pathogenicity. Um01690 protein sequence shows high similarity to SsPele1 with conserved secretion signal peptide and C‐terminal Pep1 like domain (Fig. 6a). Thus, we renamed Um01690 to UmPele1. We then tested whether UmPele1 interacts with ZmPEPR1 using Co‐IP assay in *N. benthamiana*. As shown in Fig. 6(b), ZmPEPR1‐FLAG was co‐purified with YFP‐UmPele1 but not with YFP control, showing that UmPele1 interacts with ZmPEPR1 *in planta*.

We then assessed whether UmPele1 suppresses ZmPEPR1‐mediated immunity. ZmPep1 was synthesized and used to activate immunity in maize protoplasts. We also synthesized the 25 amino acid peptide of the UmPele1 C‐terminal Pep1 like domain (highlighted by red triangle in Fig. 6(a), named as UmPel25). In maize protoplasts, co‐application of UmPel25 partially suppressed ZmPep1‐induced phosphorylation of ZmMAPK (Fig. 6c). We conclude that like the *S*. *scitamineum* effector SsPele1, *U. maydis* effector UmPele1 promotes fungal virulence at least partly by interacting with ZmPEPR1 and inhibiting activation of ZmPEPR1.

## Discussion

**Fig. 7:**
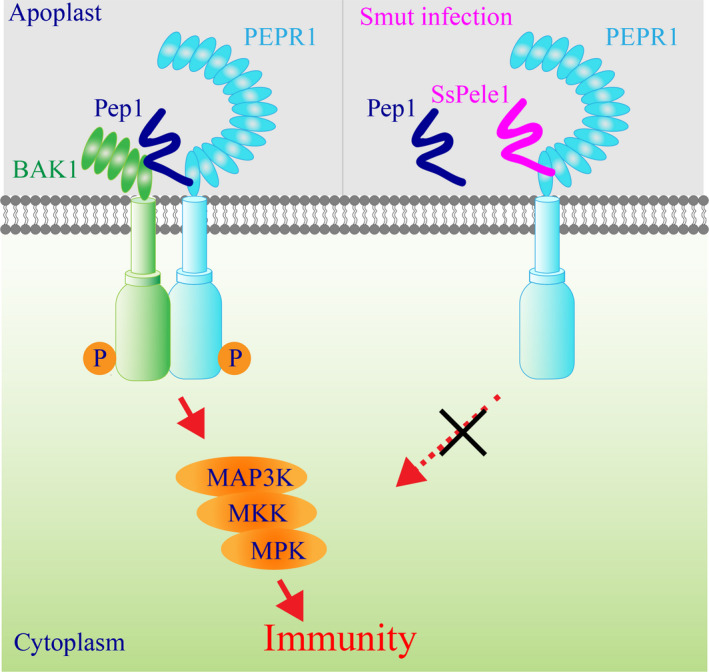
A model for the suppression of the PLANT ELICITOR PEPTIDE RECEPTOR1 (PEPR1)‐signaling by the smut effector SsPele1. The plant peptide1 (Pep1) perception induces heterodimerization and transphosphorylation of receptor kinases PEPR1 and the co‐receptor BAK1 (Bri1‐associated kinase 1). Then the activated PEPR1–BAK1 complexes induce the activation of mitogen‐activated protein kinase (MAPK) cascades through a series of phosphorylation events, resulting in the activation of PAMP‐triggered‐immunity (PTI). During the infection, the smut fungus *Sporisorium scitamineum* delivers effector SsPele1 to host apoplast, where the SsPele1 competitively binds to the extracellular domain of PEPR1 to suppress PEPR1‐mediated immune responses.

Here we show that the smut fungal effector SsPele1 and its ortholog UmPele1 contain a plant elicitor peptide‐like motif in its C‐terminus (Fig. 3e), by which SsPele1 interacts with the extracellular leucine‐rich repeat (LRR) domain of sugarcane PLANT ELICITOR PEPTIDE RECEPTOR1 (ScPEPR1), and completes ScPep1 perception, resulting in the inhibition of ScPEPR1‐mediated defense responses (Fig. 7). This reveals a novel mechanism whereby a pathogenic fungal effector simulates a nonfunctional host‐endogenous signal peptide to suppress plant defense responses. Our work also contributes a first mechanistically study on a sugarcane smut fungal effector.

Plant PEPRs are LRR kinases and receptors for endogenous peptides (Peps) (Yamaguchi *et al*., 2006, 2010; Ross *et al*., 2014; Tang & Zhou, 2016; Xu *et al*., 2018). The PEPR immune signaling is engaged in PTI and is required for systemic acquired resistance in Arabidopsis and tomato (Huffaker *et al*., 2006; Yamaguchi *et al*., 2010; Ross *et al*., 2014; Yamada *et al*., 2016; Xu *et al*., 2018). In tomato, a PEPR1 ortholog is required for systemin‐mediated resistance to the necrotrophic fungus *Botrytis cinerea* (Xu *et al*., 2018). In maize (*Zea Mays*), ZmPep1 induces the expression of defense‐related genes, the accumulation of plant defense related hormones, and resistance to pathogens (Huffaker *et al*., 2011). We found that the overexpression of *ScPEPR1* in Arabidopsis enhanced resistance to the biotrophic fungal pathogen and promoted the expression of the defense‐related genes *AtWRKY33* and *AtPR5* (Fig. S3), indicating that ScPEPR1 is a positive regulator in plant immunity. The targeting of ScPEPR1 by the effector SsPele1 indicates its importance in resistance to smut fungi. Further genetic studies, such as the overexpression or knock‐down of *ScPEPR1* in sugarcane, would illustrate the biological importance of *ScPEPR1* in resistance to smut disease.

*Ustilago maydis* effector UmPele1, an ortholog of SsPele1, has been shown to be required for *U. maydis* infection in maize (Schilling *et al*., 2014). Here, we found that SsPele1 directly interacted with ScPEPR1 (Figs 2, 3), and UmPele1 interacted with ZmPEPR1 (Fig. 6). With a functional secretion signal, SsPele1 is a secreted protein and interacts with the extracellular LRR domain of ScPEPR1 to interfere with ScPep1‐mediated defense in apoplasts (Figs 3, S5). Moreover, SsPel25 could promote growth of *Sporisorium scitamineum* on sugarcane sheath (Fig. S7). Thus, as a conserved smut fungal effector, SsPele1 contributes to the virulence of *S. scitamineum*.

Interestingly, the C‐terminus of SsPele1 and UmPele1 show a certain sequence similarity to several known plant Peps (Fig. 3d). As the extracellular domains of cell surface receptors function as interaction platforms and regulatory modules of receptor activation (Jaillais *et al*., 2011; Belkhadir *et al*., 2014), we investigated the biological relevance between ScPEPR1^LRR^ and SsPele1 using a well‐established reporter system in Arabidopsis protoplasts (Yoo *et al*., 2007). We found that ScPep1‐, but not SsPele1‐, was perceived by ScPEPR1 to induce immune responses (Figs 3e,f, 4a,b). Besides, SsPel25 competes with ScPep1 to bind to ScPEPR1 and inhibits ScPep1‐induced immune responses (Fig. 4). Furthermore, transgenic Arabidopsis overexpressing *SsPele1* exhibited reduced AtPep1‐induced early immune responses (Fig. 5). Taken together, these results allow us to present a working model for SsPele1 as shown in Fig. 7: During *S. scitamineum* infection, SsPele1 is induced and delivered to sugarcane apoplasts, where it competes with endogenous ScPep1 to interact with ScPEPR1, inhibiting ScPEPR1‐mediated immune responses.

It is interesting that both ScPep1 and SsPele1 interact with ScPEPR1; however, ScPep1 triggers the activation of ScPEPR1 signaling, whereas SsPele1 does not. The underlying mechanisms remain elusive. In Arabidopsis, AtPep1 perception by AtPEPR1 leads to the stable association of AtPEPR1 with the co‐receptor AtBAK1 (Bri1‐associated kinase 1), eliciting immune responses. Biochemical assays showed that AtPep1 induces the heterodimerization of the extracellular domains of PEPR1^LRR^‐BAK1^LRR^ (Tang *et al*., 2015). In addition to AtBAK1, another small LRR‐receptor kinase, AtAPEX interacts with AtPEPR1/2^LRR^ in a ligand‐independent manner and is required for appropriate Pep2‐induced responses (Smakowska‐Luzan *et al*., 2018). Whether SsPele1 affects the heterodimerization of ScPEPR1‐ScBAK1 or ScPEPR1‐ScAPEX is worth testing in future studies.

The extracellular space (apoplast) of plant tissue is an important battleground between plants and pathogens. Pathogenic microbes secrete apoplastic (extracellular) as well as cytoplasmic (intracellular) effectors to alter host‐cell structure and function, thereby enhancing plant susceptibility (Wawra *et al*., 2012). Apoplastic effectors mostly have been reported to function as inhibitors of proteases, chitinases, or glucanases to prevent the release of fungal elicitors (Lanver *et al*., 2017). LysM effectors are widely used by pathogenic fungi to bind to soluble chitin oligomers that could be recognized by plant immune receptors, to prevent the enzymatic hydrolyzation of host chitinase (Mentlak *et al*., 2012; Zeng *et al*., 2020). The maize smut fungus *U. maydis* secretes the effector Pit2, which inhibits a set of apoplastic papain‐like cysteine proteases by its conserved 14‐aa motif and prevents the release of endogenous SA‐associated plant defense (Mueller *et al*., 2013; Misas Villamil *et al*., 2019). The work described here show that the *S. scitamineum* apoplastic effector SsPele1, and its orthologs UmPele1 from *U. maydis*, prevent the activation of the host plant DAMP receptor ScPEPR1/ZmPEPR1 by mimicking its ligand. Besides UmPele1, the orthologs of SsPele1 were found in related smut species (Fig. 3b). It is reasonable to speculate that such a virulent strategy used by SsPele1 might be common for smut pathogens. The intracellular kinase domain of PRRs has been shown to be targeted and suppressed by pathogen effectors (Heese *et al*., 2007; Shan *et al*., 2008; Zhou *et al*., 2014). Here, our findings illustrate that the extracellular ligand‐binding domain of ScPEPR1 is targeted by SsPele1, uncovering a novel virulence mechanism for fungal apoplastic effectors.

## Author contributions

HC and YQ designed and supervised the research; HL and NH carried out the pathogen treatment, analysis of microarray data, gene cloning, Y2H, sequence alignment, qRT‐PCR, BiFC, Arabidopisis transformation, and vector construction; XF performed the protein blot, ROS, Luciferase and CoIP assays; ZZ performed the yeast secretion trial; WS performed picture capture on the laser scanning confocal microscope; and HL, HC, NH, WL and YQ wrote the manuscript. HL, XF and NH contributed equally to this work.

## Supporting information


**Fig. S1** Fungal biomass accumulation and microarray hybridization in the sugarcane‐*S*. *scitamineum* interaction.
**Fig. S2** The expression of 14 selected differentially expressed genes revealed by microarray hybridization and qRT‐PCR.
**Fig. S3** The phylogenetic analysis of PEPR1 and the expression of *ScPEPR1* in sugarcane.
**Fig. S4** Overexpression of *ScPEPR1* in Arabidopsis enhances plant resistance to powdery mildew.
**Fig. S5** SsPE14‐Δsp lacking signal peptide does not interact with ScPEPR1 in bimolecular fluorescence complementation assay.
**Fig. S6** The alignment of the amino acid sequences of plant elicitor peptides and the fungal homologs of SsPele1.
**Fig. S7** SsPel25 promotes the propagation of smut fungus on the sugarcane sheath tissue.
**Table S1** The primers and constructs used in the present study.
**Table S2** Screening genes as the detection targets of sugarcane‐*S*. *scitamineum* customization microarray.
**Table S3** The accession number of genes and proteins used in the present study.
**Table S4** Twenty‐night candidate secreted effector protein genes coexpressed with *ScPEPR1* gene.
Please note: Wiley Blackwell are not responsible for the content or functionality of any Supporting Information supplied by the authors. Any queries (other than missing material) should be directed to the *New Phytologist* Central Office.

## Data Availability

All the generated and analyzed data from this study are included in the published article and its Supporting Information.
