# Supplementary material for: A sugarcane smut fungus effector simulates the host endogenous elicitor peptide to suppress plant immunity
Source: New Phytol. 2021 Nov 12;233(2):919–33. doi: 10.1111/nph.17835 (PMC9298926; doi:10.1111/nph.17835)
Supplement: Supplementary file 1 — Fig. S1 Fungal biomass accumulation and microarray hybridization in the sugarcane‐S. scitamineum interaction. Fig. S2 The expression of 14 selected differentially expressed genes revealed by microarray hybridization and qRT‐PCR. Fig. S3 The phylogenetic analysis of PEPR1 and the expression of ScPEPR1 in sugarcane. Fig. S4 Overexpression of ScPEPR1 in Arabidopsis enhances plant resistance to powdery mildew. Fig. S5 SsPE14‐Δsp lacking signal peptide does not interact with ScPEPR1 in bimolecular fluorescence complementation assay. Fig. S6 The alignment of the amino acid sequences of plant elicitor peptides and the fungal homologs of SsPele1. Fig. S7 SsPel25 promotes the propagation of smut fungus on the sugarcane sheath tissue. Table S1 The primers and constructs used in the present study. Table S2 Screening genes as the detection targets of sugarcane‐S. scitamineum customization microarray. Table S3 The accession number of genes and proteins used in the present study. Table S4 Twenty‐night candidate secreted effector protein genes coexpressed with ScPEPR1 gene. Please note: Wiley Blackwell are not responsible for the content or functionality of any Supporting Information supplied by the authors. Any queries (other than missing material) should be directed to the New Phytologist Central Office. [file NPH-233-919-s001.pdf]

# A sugarcane smut fungus effector simulates the host endogenous elicitor peptide to suppress plant immunity

Hui Ling[1, 2#], Xueqin Fu[1#], Ning Huang[2#], Zaofa Zhong[1], Weihua Su[1], Wenxiong Lin[1], Haitao Cui[1]*, Youxiong Que[1]*

[1]Key Laboratory of Sugarcane Biology and Genetic Breeding, Ministry of Agriculture; Key Laboratory of Ministry of Education for Genetics, Breeding and Multiple Utilization of Crops; Plant Immunity Center; College of Life Sciences, Fujian Agriculture and Forestry University, Fuzhou 350002, China

[2]College of Agriculture, Yulin Normal University, Yulin 537000, China

# These authors contributed equally to this work.

* Correspondence:

Youxiong Que：+86 139 5029 5399, queyouxiong@126.com

Haitao Cui: +86 131 10898183, cui@fafu.edu.cn

Article acceptance date: 22 October 2021

## Supplementary Figures

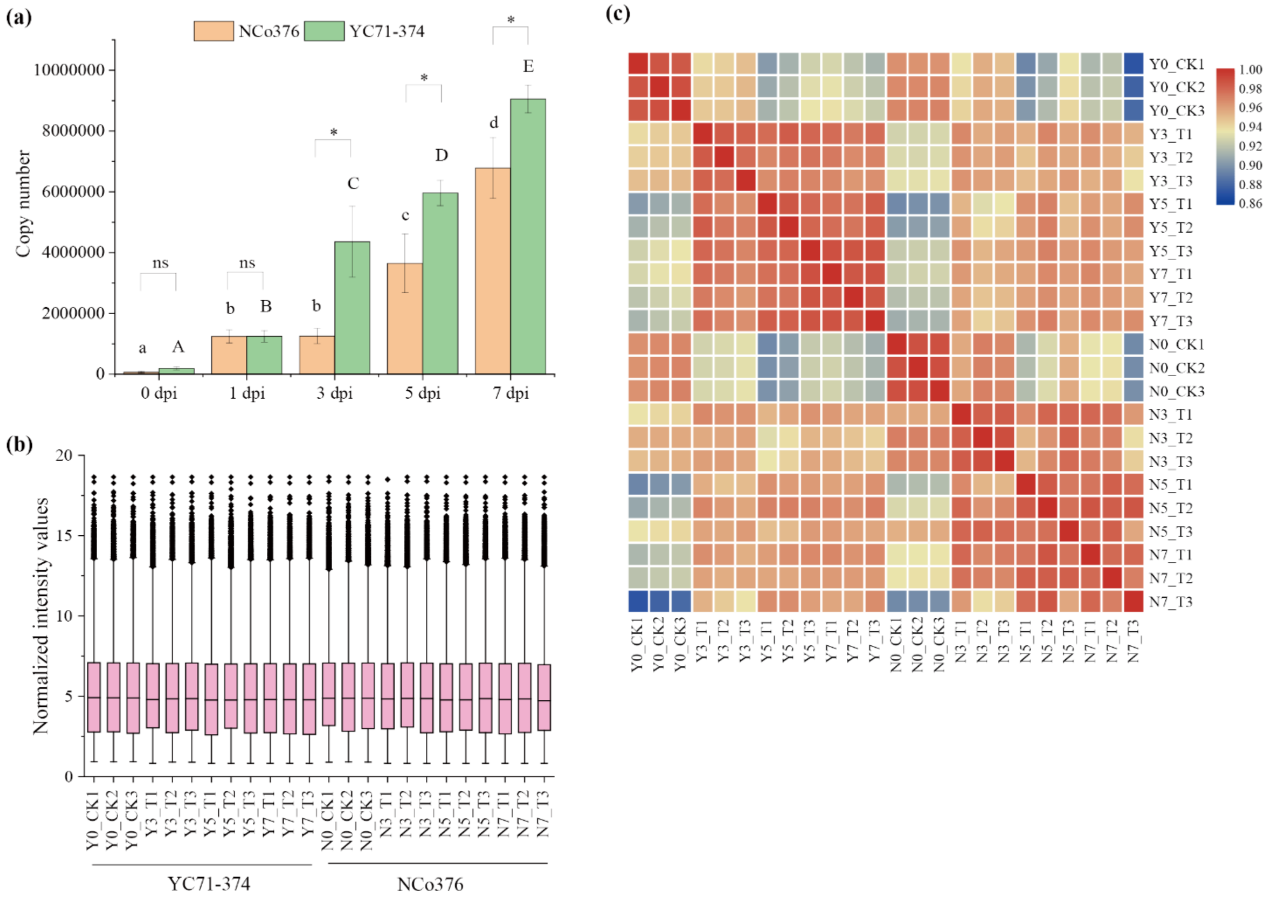


CK: Control replicate T: Treatment replicate

**Fig. S1 Fungal biomass accumulation and microarray hybridization in the sugarcane-*S. scitamineum* interaction**

(a) The accumulation of fungal biomass (copy number of fungal cells) in the resistance genotype NCo376 and the susceptible genotype YC71-374 infected with *S. scitamineum* at 0, 1, 3, 5, and 7 dpi using TaqMan based qPCR. The different letters in lowercase or uppercase indicate the significant difference of fungal biomass accumulation at different time point in Nco376 or in YC71-374 respectively ($P < 0.05$). ns, no significant difference between groups; *, the significant difference between groups ($P < 0.01$).

(b) The normalized intensity value of the expression of all targeted genes in 24 microarray samples as illustrated by the boxplot. Horizontal line indicates different samples N/Y0, 3, 5, and 7, the 0 dpi, 3 dpi, 5 dpi, and 7 dpi from NCo376 or YC71-374. CK/T1, 2, and 3, the biological replicate 1, 2, and 3 of the control or the treatment of *S. scitamineum*. The normalized intensity value of gene expression was achieved from the normalization and log-transformation of microarray hybridization signaling value. The whiskers represent the gene expression level with values within 1.5 times the median. Black dots beyond the whiskers are the individual values out of 1.5 time the interquartile range with outliers shown as fliers.

(c) Pearson's correlation analysis of the 24 microarray samples based on the normalized gene expression data. The table cell is color-coded by correlation according to the color legend. CK/T1, 2, and 3, the biological replicate 1, 2, and 3 of the control or the treatment of *S. scitamineum*.

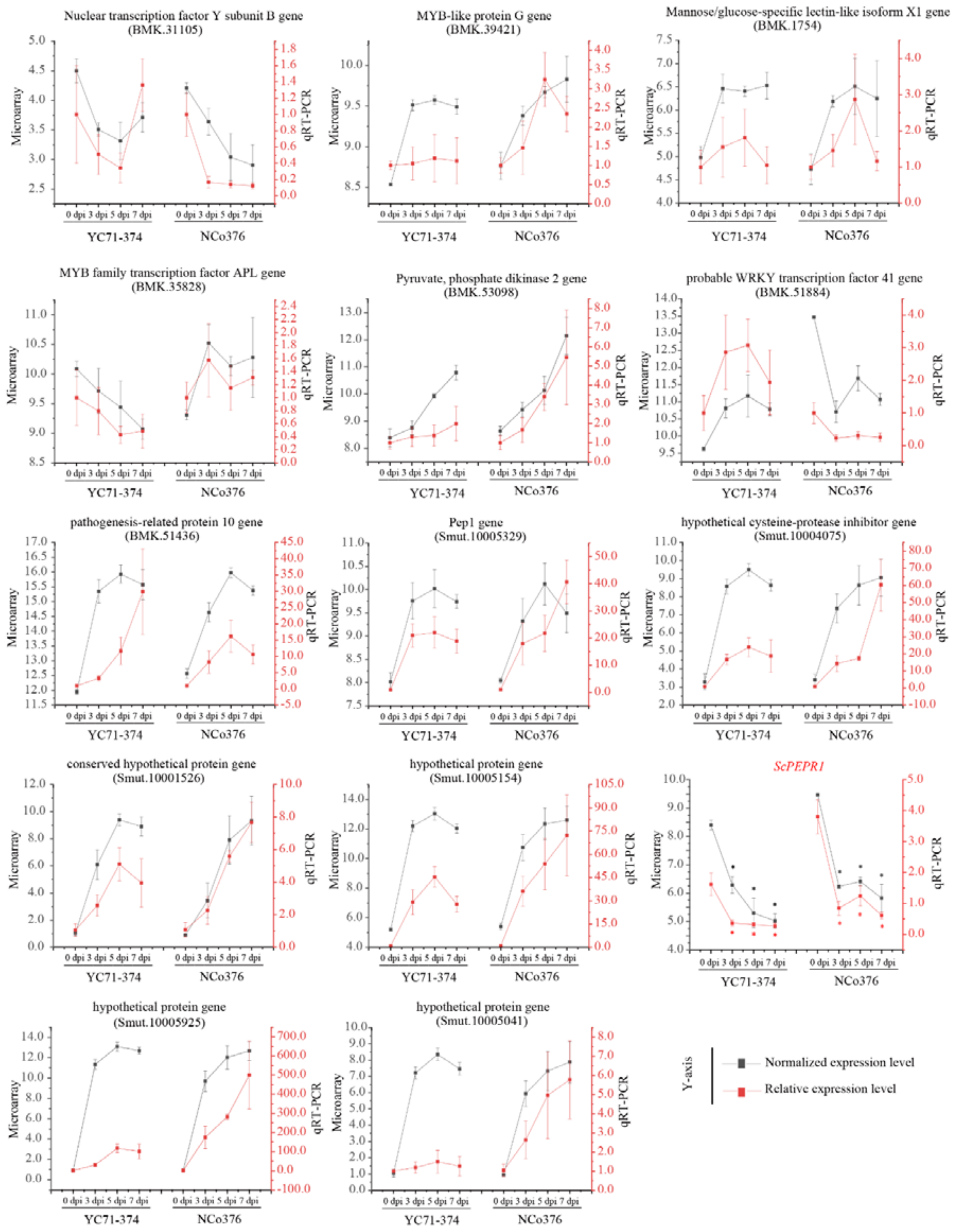


**Fig. S2 The expression of 14 selected DEGs revealed by microarray hybridization and qRT-PCR**

Based on the DEGs (differentially expressed genes) from the microarray hybridization, 8 DEGs from sugarcane and 6 DEGs from *S. scitamineum* were selected, and the gene expression was validated in the smut-susceptible genotype YC71-374 and smut-resistant genotype NCo376 using qRT-PCR. The accession number under the functional annotation of gene indicates a gene from sugarcane (BMK.xxx) or *S. scitamineum* (Smut.xxx). The error bars indicate standard deviation (n=3). * indicates a significant difference between the 0 dpi and 3, 5, or 7 dpi ($P < 0.05$) determined by Student's *t* test.

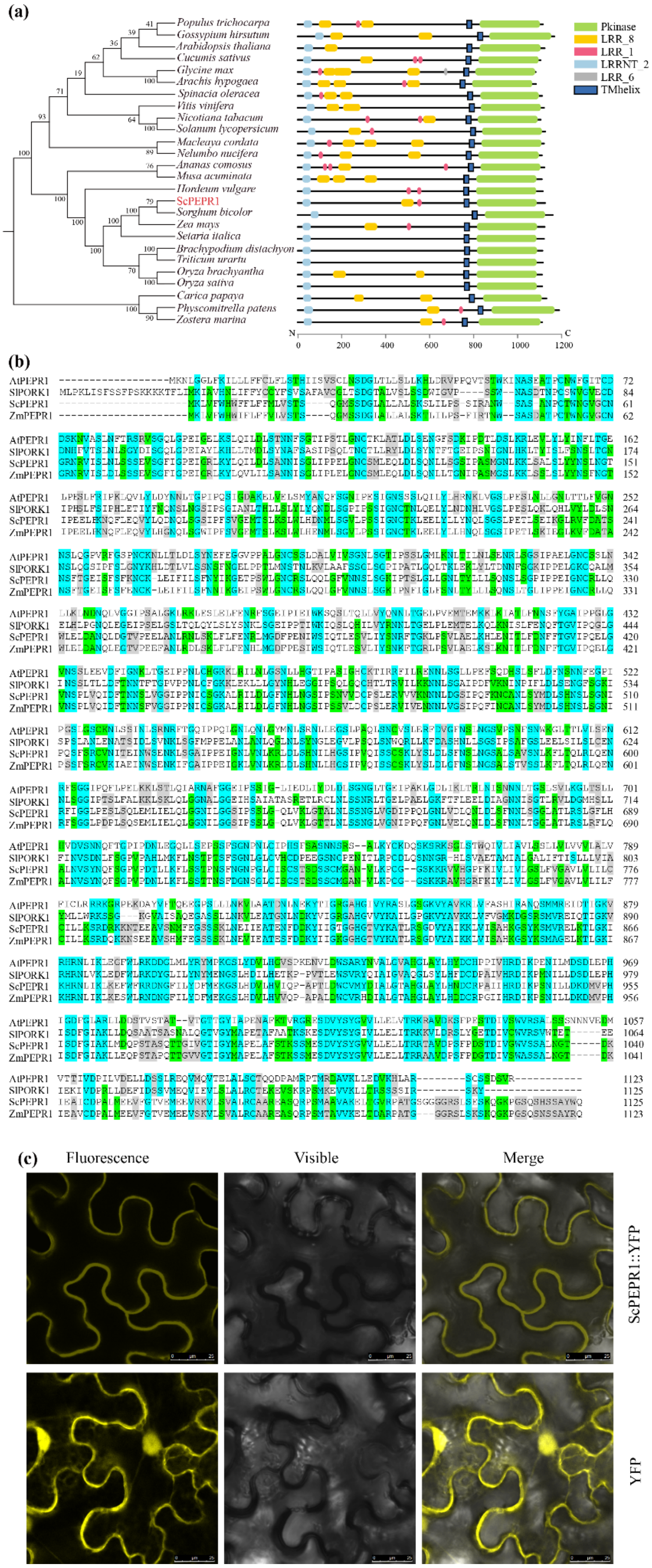


**Fig. S3 The phylogenetic analysis of PEPR1 and the expression of *ScPEPR1* in sugarcane**

(a) Phylogenetic and conserved domain analysis of ScPEPR1. The ScPEPR1 orthologs were obtained from *Sorghum bicolor*, *Zea mays*, *Setaria italic*, *Brachypodium distachyon*, *Oryza brachyantha*, *Oryza sativa* Japonica, *Triticum urartu*, *Hordeum vulgare*, *Ananas comosus*, *Musa acuminate*, *Macleaya cordata*, *Vitis vinifera*, *Nelumbo nucifera*, *Nicotiana tabacum*, *Spinacia oleracea*, *Populus trichocarpa*, *Solanum lycopersicum*, *Gossypium hirsutum*, *Glycine max*, *Arabidopsis thaliana*, *Arachis hypogaea*, *Cucumis sativus*, *Physcomitrella patens*, *Carica papaya*, and *Zostera marina*. The rectangle with different colors represents different conserved protein domain. The accession number can be found in the table S3. Numbers at the branches indicate bootstrap values.

(b) The protein alignment of plant PEPR. ScPEPR1 and its orthologs from *Arabidopsis thaliana*, *Zea mays*, and *Solanum lycopersicum* were aligned using DNAMAN and Genedoc, and the low homologous sites were removed. The highly conserved residues were marked with color. The accession number of the sequences used in the present study can be found in table S3.

(c) ScPEPR1 is located on the cytomembrane. YFP and ScPEPR1-YFP were expressed in *N. benthamiana* leaves. The protein subcellular localization was observed using a confocal microscope 2 d after *Agrobacterium tumefaciens* transformation. Bar scale = 5 μm.

These experiments were repeated at least three times with consistent results.

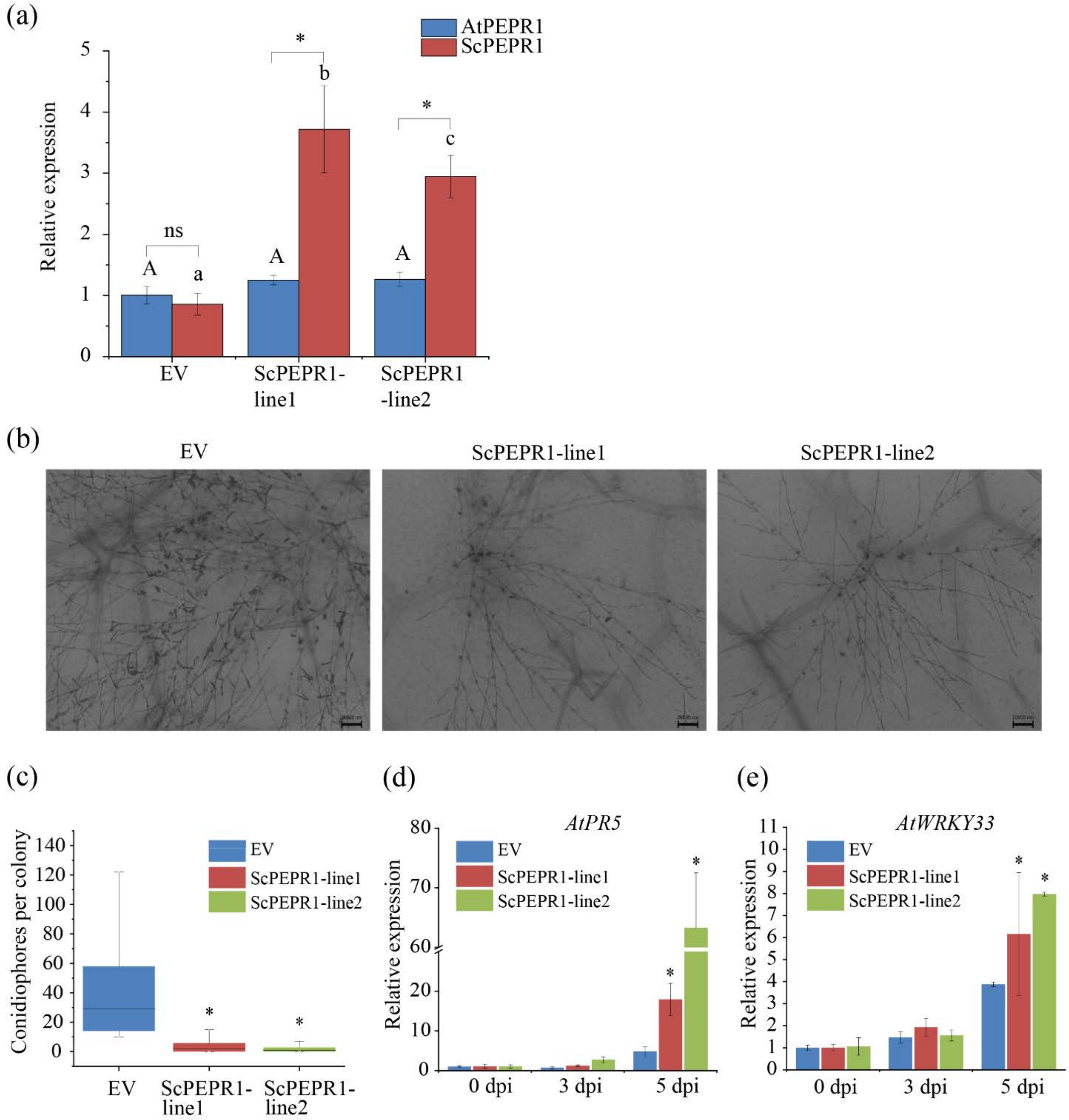


**Fig. S4 Overexpression of *ScPEPR1* in *Arabidopsis* enhances plant resistance to powdery mildew**

(a) The expression of *ScPEPR1* and *AtPEPR1* in *ScPEPR1* transgenic lines. The bars indicate relative fold change ± SD (n=3) among bio-replicates. The different letters in lowercase or uppercase indicate the significant difference of the gene expression in different lines ($P < 0.01$). ns, no significant difference between two genes in the same line; *, the significant difference between two genes in the same line ($P < 0.01$).

(b) Trypan blue staining of hyphae and conidiophores on leaves of empty-vector (EV) transgenic lines and *ScPEPR1* transgenic lines (ScPEPR1-line1 and line2) after infection with *G. cichoracearum* at 5 dpi. Scale bar =20 um. These experiments were repeated three times with consistent results.

(c) Quantification of fungal growth on *ScPEPR1* transgenic *Arabidopsis* line1/2 and empty-vector (EV) transgenic line at 5 dpi by counting the number of conidiophores per colony of the powdery mildew fungus *G. cichoracearum*. Bars represent mean and SD (n >= 68).

(d-e) The expression of the defense-related marker gene *AtWRKY33* and the SA-dependent marker gene *AtPR5* on *ScPEPR1* transgenic line1/2 and empty-vector (EV) transgenic line at indicated time after infection by *G. cichoracearum*. The bars indicate relative fold change ± SD (n=3) compared to EV line at 0 dpi. In a-c, * indicates a significant difference compared to the empty-vector (EV) transgenic line ($P< 0.05$) determined by Student’s *t* test.

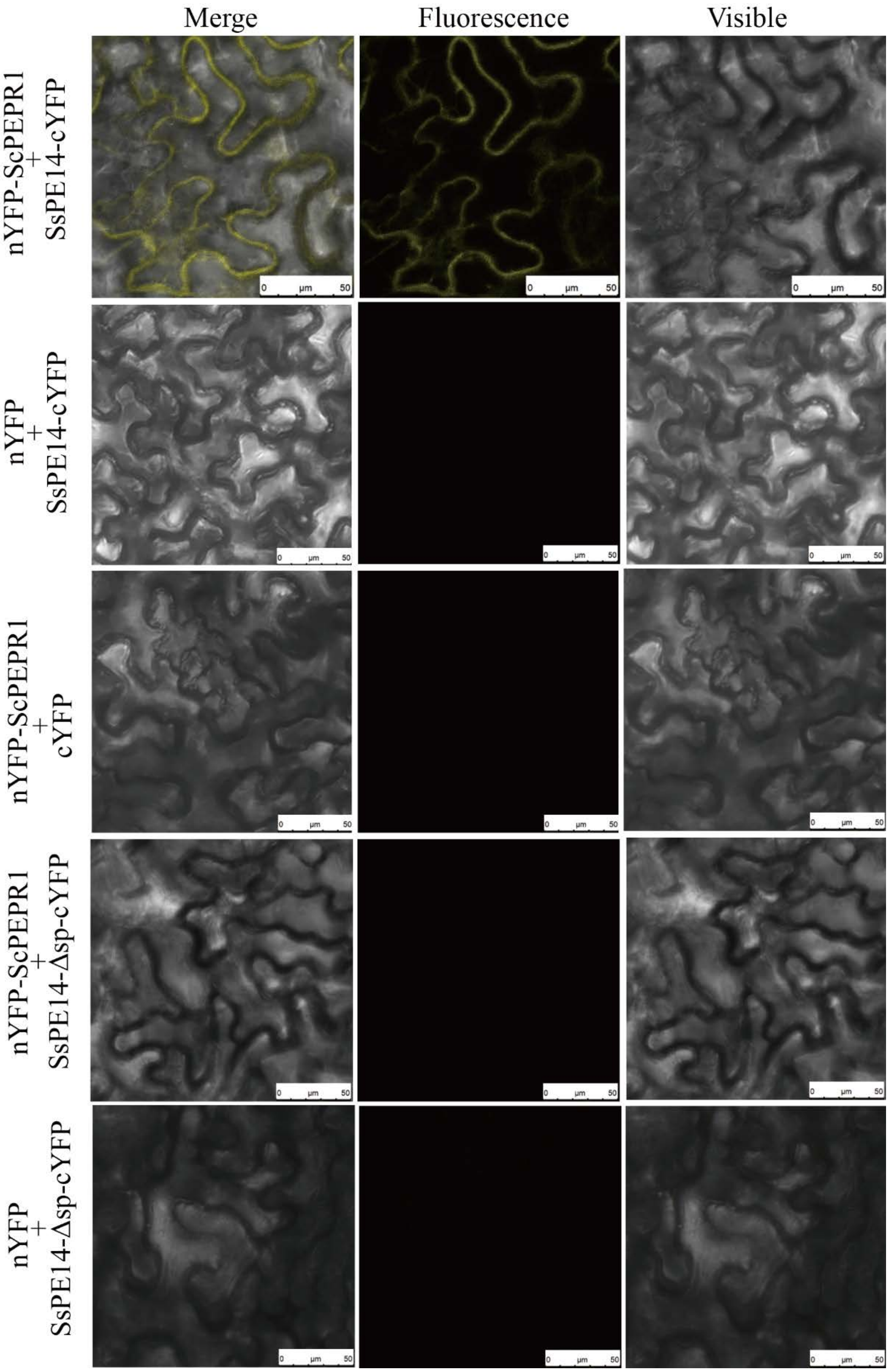


**Fig. S5 SsPE14-Δsp lacking signal peptide does not interact with ScPEPR1 in BiFC assay**

The binding assay between ScPEPR1 and SsPE14-Δsp in *Nicotiana benthamiana*, revealed by BiFC. The *N. benthamiana* leaves were co-infiltrated with *35S:nYFP-ScPEPR1* and *35S:SsPE14-cYFP*, *or 35S:SsPE14*-Δsp-*cYFP*. Images were captured by a confocal microscope at 2 d after *Agrobacterium tumefaciens* transformation. SsPE14-Δsp, a signal peptides deletion mutant of SsPE14. Scale bars, 10 μm.

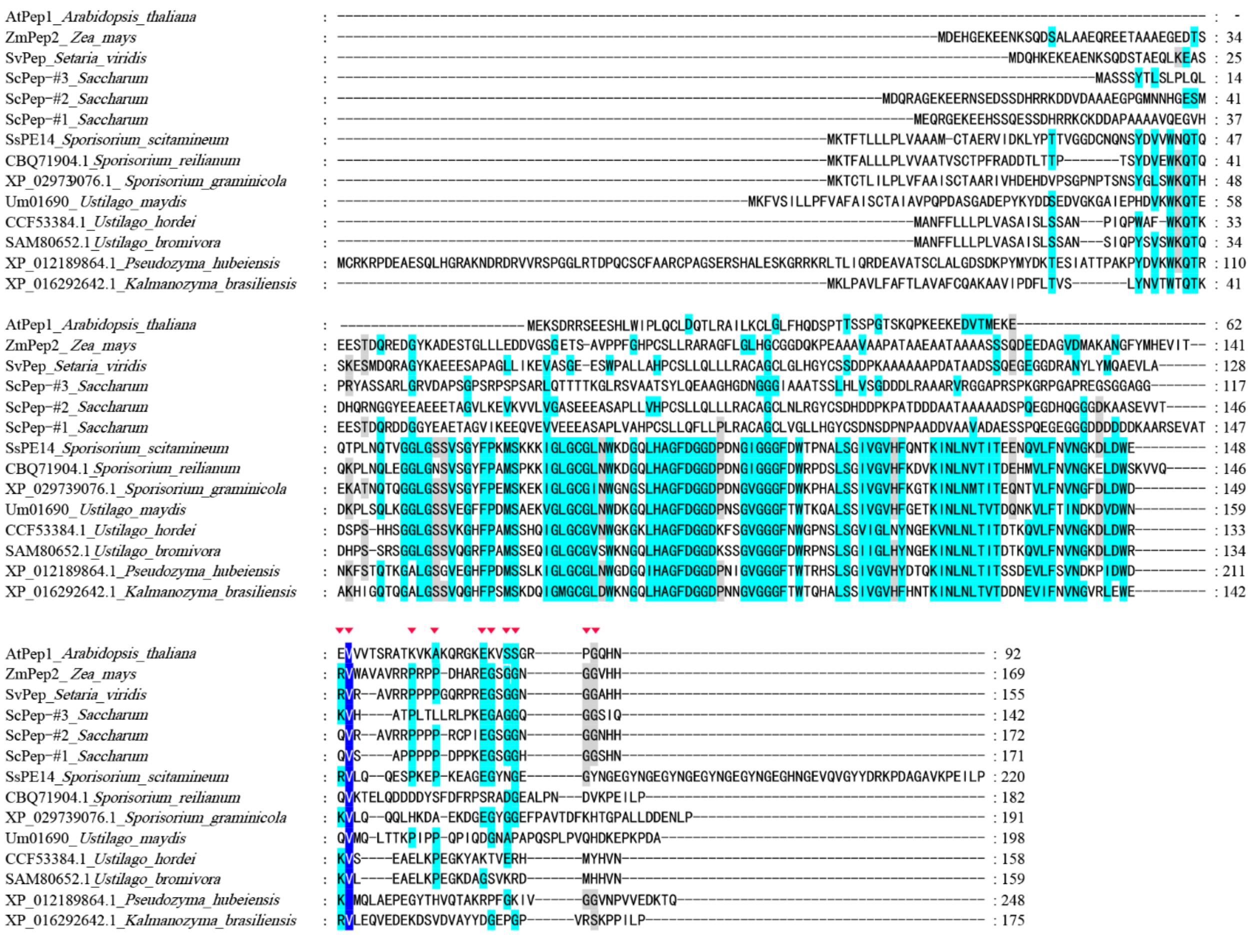


**Fig. S6 The alignment of the amino acid sequences of plant elicitor peptides and the fungal homologs of SsPele1**

The Pep1 protein sequences of Arabidopsis AtPep1, maize ZmPep2, green foxtail SvPep and sugarcane ScPep-#1/#2/#3 and the Pele1 protein sequences from *Sporisorium reilianum*, *Sporisorium graminicola*, *Ustilago maydis*, *Ustilago hordei*, *Ustilago bromivora*, *Pseudozyma hubeiensis*, and *Kalmanozyma brasiliensis* obtaining from the NCBI database (Table S3) were aligned with DNAman and DNAMAN (Version 7.0.2.176) and Genedoc (http://www.flu.org.cn/en/download-47.html). The highly conserved sites are colored and marked with red triangle symbol on the top row. The accession number of the sequences used in the present study can be found in table S3.

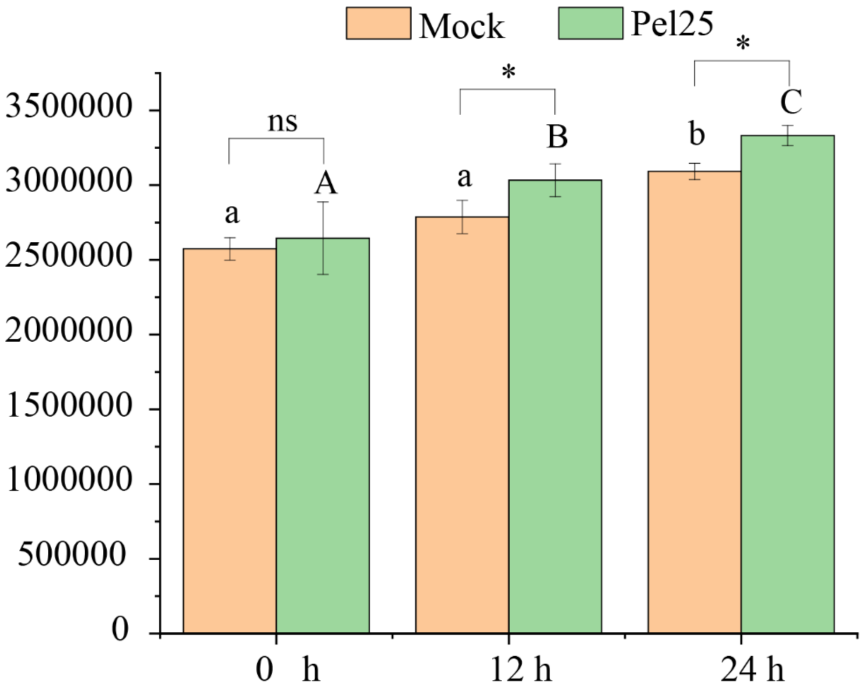


**Fig. S7 SsPel25 promotes the propagation of smut fungus on the sugarcane sheath tissue**

Sheath tissues were treated with 5 μM SsPel25, and immersed into the diploid-type smut fungus ($OD_{600}$=0.008) solution and sampled at the indicated time points. A TaqMan-based qPCR detection of the increase of the fungal biomass were conducted according to Su et al. (2013). The different letters in lowercase or uppercase indicate the significant difference of fungal biomass accumulation at different time point in the mock samples or the SsPele1 treated samples, respectively ($P$ < 0.05). ns, no significant difference between two groups; *, the significant difference between two groups ($P$ < 0.01).

## Supplementary Tables

Table S1 The primers and constructs used in the present study

| Genes | Sequences (Forward/Reverse primer) | Application |
|---|---|---|
| *bE* | TGAAAGTTCTCATGCAAGCC/TGAGAGGTCGATTGAGGTTG | Su *et al.* (2013), for Taqman based qPCR detection of smut fungus |
| *bE* | TGCTCGACGCCAATTCGGAG (probe) | Su *et al.* (2013), for Taqman based qPCR detection of smut fungus |
| *SsPele1** | ATGAAGACCTTCACGCTCCTCCTG/TCAAGGCAGTATCTCAGGCTTGACG | For cloning |
| *SsPele1* | CATATGGCCATGGAGGCCGAATTCATGAAGACCTTCACGCTCCT/GCGGCCGCTGCAGGTCGACGGATCCAGGCAGTATCTCAGGCTTGA | pGBKT7, for Y-2-H |
| *SsPele1-ΔC* | ATGGCCATGGAGGCCGAATTCATGAAGACCTTCACGCTCCT/GCGGCCGCTGCAGGTCGACGGATCCCTGGAGAACCCTCTCCCAGT | pGBKT7, for Y-2-H |
| *SsPele1* | CTCGAGGGGGGGCCCGGTACCATGAAGACCTTCACGCTCCTC/TTGAACGATCTGCAGCCCGGGTCAAGGCAGTATCTCAGGC | pCAMBIA1301, for *Arabidopsis* transformation |
| *SsPele1* | CGACTCTAGGAGCTCGGTACCCGGGATGAAGACCTTCACGCTCCTC/GGAACATCGTATGGGTACATACTAGTAGGCAGTATCTCAGGCTTGAC | pCAMBIA2300S-YC, for BiFC |
| *SsPele1* | TCGGTACCTCGCGAATACATCTAGATGGATCCATGGAACGCGTGATTGATAAAC/TGCAGTCGACGGGCCCGGGATCCGATGCGGCCGCATGGTCGGTCTCTTA | pGEX-4T-1_SsPele1, for ectopic expression in *Escherichia coli*** |
| *SsPele1* | GGGGACAAGTTTGTACAAAAAAGCAGGCTTCATGAAGACCTTCACGCTCCTCC/GGGGACCACTTTGTACAAGAAAGCTGGGTCAGGCAGTATCTCAGGCTTGACG | pDONR207, for construction of pFAST-R06-SsPele1::eGFP |
| *SsPele1* | AAGGTCTCAAATGAAGACCTTCACGCTCCTCC/AAGGTCTCACGAATTCACATTGAACAGTACCT | pXCSG-_SsPE14::eYFP, for CoIP |
| *SsPele1* | CGGAATTTTAATTAAGAATTCATGAAGACCTTCACGCTCCTC/CACTATAGGGAGAACCTCGAGGTCGCCGCCGACAGTAGT | pSUC2T7M13ori- SsPE14 |
| *SsPele1-ΔC* | ATGAAGACAAAATGAAtACCTTCACGCTCCTCC/CTGAAGACTTAAGCTCAAGGCAGTATCTCAGGCT | pXCSG-_SsPele1::eYFP, for CoIP |
| *SsPele1-Δsp* | CGACTCTAGGAGCTCGGTACCCGGGATGCGAGTCATCGACAAGCTCTA/GGAACATCGTATGGGTACATACTAGTAGGCAGTATCTCAGGCTTGAC | pCAMBIA2300S-YC, for BiFC |
| *SsPE1* | ATGATCGAGCTCACTACCGCGTTTGTCGC/TCACTTCAAAGCGGCCTGGTAAAGGATAGGCTC | For cloning |
| *SsPE1* | CATATGGCCATGGAGGCCGAATTCATGATCGAGCTCACTACC/GCGGCCGCTGCAGGTCGACGGATCCCTTCAAAGCGGCCTGGTAAA | pGBKT7, for Y-2-H |
| *SsPE4* | ATGCTGGTTCACTCAGCTCC/TCAATGAGAGCGACCCTGTTTC | For cloning |

Table S1 continued

| Genes | Sequences (Forward/Reverse primer) | Notes |
|---|---|---|
| *SsPE4* | CATATGGCCATGGAGGCCGAATTCATGCTGGTTCACTCAGCTCC/GCGGCCGCTGCAGGTCGACGGATCCATGAGAGCGACCCTGTTTCTCG | pGBKT7, for Y-2-H |
| *SsPE15* | ATGAAGTTCGCCTTTGGATCC/CTAACCCTGAGTGTTGATGTCG | For cloning |
| *SsPE15* | CATATGGCCATGGAGGCCGAATTCATGAAGTTCGCCTTTGGATC/GCGGCCGCTGCAGGTCGACGGATCCACCCTGAGTGTTGATGTCG | pGBKT7, for Y-2-H |
| *ScPEPR1* | GCGAAAACTAGAGCAGCCCC/AACAGCTCGGCAGAATCAGAA | For cloning |
| *ScPEPR1* | GCCATGGAGGCCAGTGAATTCATGAAGCTGGTTTTCTGGC/CAGCTCGAGCTCGATGGATCCCTGCCAGTACGCGCTGC | pGADT7, for Y-2-H |
| *ScPEPR1* | CGGGGGACGAGCTCGGTACCATGAAGCTGGTTTTCTGGC/TCCAAGGGCGAATTGGTCGACCTGCCAGTACGCGCTGC | pCAMBIA1306-flag, for Arabidopsis transformation |
| *ScPEPR1* | ATCACAAAGTGCATCATCATCATCATCATATGAGTAGTGATGGTCTG/CTGTGCTTTTAAGCAGAGATTACCTATCTAGATTTAAACGGACCATGCACAACGCG | pCzn1, for ectopic expression in *Escherichia coli*** |
| *ScPEPR1* | GATTTCTGAGGAGGATCTTCCCGGGATGAAGCTGGTTTTC/GCAGGGCATGCCTGCAGGTCGACCTACTGCCAGTACGC | pCMABIA1300S-YN::*ScPEPR1* |
| *ScPEPR1:* | CGAACGATACTCGAGGTCGACATGAAGCTGGTTTTCTGGC/GCTCACCATACTAGTGGATCCCTGCCAGTACGCGCTGC | pCAMBIA2300_*ScPEPR1::* YFP, for subcellular location assay |
| *ScPEPR1-N* | GCCATGGAGGCCAGTGAATTCATGAAGCTGGTTTTCTGGC/CAGCTCGAGCTCGATGGATCCTTTGAATGGGCCATGCAC | pGADT7, for Y-2-H |
| *ScPEPR1-N* | ATCACAAAGTGCATCATCATCATCATCATATGAGTAGTGATGGTCTGGC/CTGTGCTTTTAAGCAGAGATTACCTAAATTTCCGGCGGAATGGCGCCA | pCzn1, for ectopic expression in *Escherichia coli** |
| *ScPEPR1-C* | GCCATGGAGGCCAGTGAATTCATGTTGAAATCTCGAGATCGGA/CAGCTCGAGCTCGATGGATCCCTGCCAGTACGCGCTGC | pGADT7, for Y-2-H |
| *ZmPEPR1* | CGGGGGACGAGCTCGGTACCATGAAGCTGGTTTTCTGGCA/CAGCTCGAGCTCGATGGATCCCGGATTGCGATCCTGGTTTCC | pCAMBIA1306-flag, for CoIP |
| *AtPEPR1* | CGGGGGACGAGCTCGGTACCATGAAGAATCTTGGGGGGTT/TCCAAGGGCGAATTGGTCGACCCGAACTGAATCAGAGGAGC | pCAMBIA1306-flag, for immunoprecipitation |
| *AtPEPR1* | ATTCCTATTGAGATATGGAAGAG/CCTCTTCTAAGCTGCTGTTCAC | For qRT-PCR |

* *SsPele1* and *SsPE14* were the same gene.

** Codon optimization and synthetic gene coding sequence were used to enhance protein expression in *Escherichia coli* strain BL21.

Table S2 Screening genes as the detection targets of sugarcane-*S. scitamineum* customization microarray

| Category | Key word |
|---|---|
| Receptor | Receptor |
| Kinase | Kinase |
| Phosphatase | Phosphatase |
| signal transduction | Calmodulin, guanine-nucleotide-exchange etc. |
| Plant hormone | Auxin, cytokinin, ethylene, gibberellin, jasmonate, salicylate etc. |
| Transcription factor | Transcription factor, DNA binding |
| Reactive oxygen | Respiratory burst oxidase, superoxide dismutase, catalase etc. |
| Programmed cell death | senescence-associated, apoptosis, Metacaspase etc. |
| Ubiquitination | Ubiquitin |
| Resistance | Elicitor, R gene,cell wall-associated etc. |
| Energy metabolism | Glucanase, ATP, cytochrome b5 reductase etc. |

Table S3 The accession number of genes and proteins used in the present study

| Species | Gene/protein name | GenBank accession NO. |
|---|---|---|
| *Saccharum* | *ScPEPR1* | MN445188 |
| *Arabidopsis thaliana* | AtPEPR1 | NP_177451 |
| *Sorghum bicolor* | ortholog of AtPEPR1 | XP_002445581 |
| *Zea mays* | *ZmPEPR1* | PWZ26320 |
| *Setaria italic* | ortholog of AtPEPR1 | XP_012702562 |
| *Brachypodium istachyon* | ortholog of AtPEPR1 | XP_010235180 |
| *Oryza brachyantha* | ortholog of AtPEPR1 | XP_006659458 |
| *Oryza sativa* Japonica | ortholog of AtPEPR1 | XP_015648066 |
| *Triticum urartu* | ortholog of AtPEPR1 | EMS62275 |
| *Hordeum vulgare* | ortholog of AtPEPR1 | BAK08322 |
| *Ananas comosus* | ortholog of AtPEPR1 | XP_020083255 |
| *Musa acuminate* | ortholog of AtPEPR1 | XP_009383175 |
| *Macleaya cordata* | ortholog of AtPEPR1 | OVA13263 |
| *Vitis vinifera* | ortholog of AtPEPR1 | XP_002273607 |
| *Nelumbo nucifera* | ortholog of AtPEPR1 | XP_010246888 |
| *Nicotiana tabacum* | ortholog of AtPEPR1 | XP_016436237 |
| *Spinacia oleracea* | ortholog of AtPEPR1 | XP_021845333 |
| *Populus trichocarpa* | ortholog of AtPEPR1 | XP_002311912 |
| *Solanum lycopersicum* | ortholog of AtPEPR1 | XP_004235511 |
| *Gossypium hirsutum* | ortholog of AtPEPR1 | XP_016711407 |
| *Glycine max* | ortholog of AtPEPR1 | XP_003555482 |
| *Arachis hypogaea* | ortholog of AtPEPR1 | XP_025611232 |
| *Cucumis sativus* | ortholog of AtPEPR1 | XP_011651735 |
| *Physcomitrella patens* | ortholog of AtPEPR1 | XP_024359221 |
| *Carica papaya* | ortholog of AtPEPR1 | XP_021896791 |
| *Zostera marina* | ortholog of AtPEPR1 | KMZ64621 |
| *Saccharum* | plant elicitor peptide/ScPep-#3 | Cluster-44244.0 |
| *Saccharum* | plant elicitor peptide/ScPep-#2 | Sspon.05G0022590-1B |
| *Saccharum* | plant elicitor peptide/ScPep-#1 | Sspon.05G0022600-1B |
| *Zea mays* | ZmPep2 | ALF84910.1 |
| *Setaria viridis* | SvPep | TKW06853 |
| *Sporisorium scitamineum* | putative effector 1(*SsPE1*) | MK756111 |
| *Sporisorium scitamineum* | putative effector 4 (*SsPE4*) | MK756112 |
| *Sporisorium scitamineum* | putative effector 14 (SsPE14) | MK756113 |
| *Sporisorium scitamineum* | putative effector 15 (*SsPE15*) | MK756114 |
| *Sporisorium reilianum* | ortholog of SsPele1/SsPE14 | CBQ71904.1 |
| *Sporisorium graminicola* | ortholog of SsPele1/SsPE14 | XP_029739076.1 |
| *U. maydis* | ortholog of SsPele1/SsPE14 | XP_011387667.1/um01690 |
| *Ustilago hordei* | ortholog of SsPele1/SsPE14 | CCF53384.1 |
| *Ustilago bromivora*; | ortholog of SsPele1/SsPE14 | SAM80652.1 |
| *Pseudozyma_hubeiensis* | ortholog of SsPele1/SsPE14 | XP_012189864.1 |
| *Kalmanozyma_brasiliensis* | ortholog of SsPele1/SsPE14 | XP_016292642.1 |
| *Moesziomyces antarcticus* | ortholog of SsPele1/SsPE14 | XP_014658179.1 |

Table S4 Twenty-nine candidate secreted effector protein genes coexpressed with sugarcane *plant elicitor peptide receptor1 gene*

| | Gene ID* | Genbank accession NO. | Functional Annotation |
|---|---|---|---|
| 1 | Smut.1000502 | MK756113 | plant elicitor peptide-like effector1 |
| 2 | Smut.10000247 | CBQ71258.1 | hypothetical protein |
| 3 | Smut.10000198 | CBQ71336.1 | hypothetical protein |
| 4 | Smut.10004248 | XP_757351.1 | hypothetical protein UM01204.1 |
| 5 | Smut.10002383 | CBQ72682.1 | hypothetical Ustilaginaceae-specific protein |
| 6 | Smut.10001535 | CBQ70070.1 | hypothetical Ustilaginaceae-specific protein |
| 7 | Smut.10002181 | CBQ68784.1 | probable alpha-galactosidase |
| 8 | Smut.10001520 | CBQ70098.1 | hypothetical protein |
| 9 | Smut.10000668 | CBQ69161.1 | related to fruiting body protein SC7 precursor |
| 10 | Smut.10002097 | CBQ68888.1 | hypothetical protein |
| 11 | Smut.10004684 | CBQ70535.1 | hypothetical protein |
| 12 | Smut.10005166 | CBQ72054.1 | related to α-L-arabinofuranosidase I precursor |
| 13 | Smut.10000851 | CBQ68954.1 | hypothetical protein |
| 14 | Smut.10004073 | CBQ70787.1 | hypothetical protein |
| 15 | Smut.10002573 | CBQ72520.1 | probable Endoglucanase 1 precursor (egl1) |
| 16 | Smut.10001004 | CBQ69118.1 | related to acetylxylan esterase |
| 17 | Smut.10005925 | CBQ67365.1 | hypothetical protein |
| 18 | Smut.10000512 | CBQ69902.1 | hypothetical protein |
| 19 | Smut.10002386 | CBQ72684.1 | hypothetical Ustilaginaceae-specific protein |
| 20 | Smut.10005024 | CBQ71904.1 | hypothetical protein |
| 21 | Smut.10002384 | CBQ72682.1 | hypothetical Ustilaginaceae-specific protein |
| 22 | Smut.10000388 | CBQ69595.1 | hypothetical protein |
| 23 | Smut.10004075 | CBQ70785.1 | hypothetical protein |
| 24 | Smut.10005136 | CBQ72022.1 | probable protein disulfide-isomerase precursor |
| 25 | Smut.10001934 | CBQ73920.1 | hypothetical protein |
| 26 | Smut.10006601 | GAC98548.1 | hypothetical protein PHSY_006142 |
| 27 | Smut.10005041 | CBQ71923.1 | hypothetical protein |
| 28 | Smut.10003210 | CBQ73627.1 | related to Mig1 protein |
| 29 | Smut.10005154 | CBQ72040.1 | hypothetical protein |

* Gene ID was achieved from the genomic sequencing data Que et al. (2014a).

## Reference

**Su Y, Wang S, Guo J, Xue B, Xu L, Que Y. 2013.** A TaqMan real-time PCR assay for detection and quantification of *Sporisorium scitamineum* in sugarcane. *Scientific World Journal* **2013**(9): 942682.

**Que Y, Xu L, Wu Q, Liu Y, Ling H, Liu Y, Zhang Y, Guo J, Su Y, Chen J. 2014a.** Genome sequencing of *Sporisorium scitamineum* provides insights into the pathogenic mechanisms of sugarcane smut. *BMC Genomics* **15**(1): 996.
